# Influenza A (H1N1)pdm infection exacerbates behavioral, cognitive, and synaptic impairments in 5xFAD mice

**DOI:** 10.3389/fnbeh.2026.1955816

**Published:** 2026-10-08

**Authors:** Anastasiya Rakovskaya, Alexey Lozhkov, Valeria Tverdokhlebova, Yana Zabrodskaya, Egor Vinokurov, Valeria Kirenskaya, Alexander Erofeev, Angelika Garshinina, Valeria Zryacheva, Anna Shtro, Ekaterina Pchitskaya, Olga Vlasova, Ekaterina Elpaeva, Pavel Dav, Ilya Bezprozvanny, Andrey Vasin

**Affiliations:** 1 Institute of Biomedical Systems and Biotechnology, Peter the Great St. Petersburg Polytechnic University, St. Petersburg, Russia; 2 Smorodintsev Research Institute of Influenza, St. Petersburg, Russia; 3 Pavlov Institute of Physiology, Russian Academy of Sciences, St. Petersburg, Russia; 4 National Medical Research Center of Oncology named after N.N. Petrov, St. Petersburg, Russia

**Keywords:** Alzheimer’s disease, glial reactivity, influenza virus, neuroinflammation, synaptic plasticity

## Abstract

Alzheimer’s disease (AD) is characterized by progressive cognitive decline, synaptic dysfunction, and neuroinflammation, which may be influenced by systemic factors such as infection. The present study aimed to investigate the effects of prior influenza A (H1N1)pdm infection on behavioral, synaptic, and neuroinflammatory alterations in the 5xFAD mouse model of AD. Mice were intranasally infected at 2 months of age and underwent behavioral assessment from 7 months of age using tests of social, affective, exploratory, and cognitive function. Hippocampal synaptic function and structure were assessed by CA1 long-term potentiation (LTP) recordings and dendritic spine analysis, while neuroinflammation and amyloid pathology were evaluated by Western blot and immunohistochemistry. 5xFAD mice exhibited impairments in hippocampus-dependent memory, social behavior, and affective responses. Prior H1N1pdm infection modulated these phenotypes, resulting in increased depressive-like behavior in the tail suspension test and alterations in exploratory and social activity. At the synaptic level, reduced LTP and decreased dendritic spine density were observed in 5xFAD mice, along with a shift toward immature spine morphology. Viral infection induced similar synaptic alterations in wild-type mice but did not further exacerbate synaptic deficits in 5xFAD animals. Both amyloid pathology and infection were associated with increased astrocytic and microglial activation, with the strongest neuroinflammatory response detected in infected 5xFAD mice. Overall, prior H1N1pdm infection altered behavioral and neuroinflammatory phenotypes but did not further aggravate the already established synaptic deficits in 5xFAD mice. These findings support a non-additive interaction between systemic inflammation and neurodegenerative pathology and may help explain the heterogeneous consequences of viral infection in older individuals with cognitive impairment.

## 1 Introduction

Alzheimer’s disease (AD) is a progressive neurodegenerative disorder characterized by cognitive decline, synaptic dysfunction, and neuronal loss. Its classical neuropathological hallmarks include extracellular β-amyloid (Aβ) plaques and intracellular neurofibrillary tangles, accompanied by widespread synaptic pathology (Scheff et al., 2006; DeTure and Dickson, 2019). Beyond protein aggregation, neuroinflammation and dysregulated intracellular calcium signaling are increasingly recognized as important contributors to disease progression (Heneka et al., 2015; Nesterova et al., 2019; Bezprozvanny and Mattson, 2008; Bezprozvanny, 2010). Microglia and astrocytes are central regulators of these inflammatory responses and can influence synaptic integrity and neuronal survival (Leng and Edison, 2021; Hong et al., 2016; Colombo and Farina, 2016).

Synaptic loss is among the earliest and strongest correlates of cognitive decline in AD. Alterations in dendritic spine density and morphology, particularly a shift from mature mushroom spines toward thin and stubby phenotypes, are associated with impaired synaptic stability and plasticity (Shankar et al., 2007; Spires-Jones and Hyman, 2014). Calcium-dependent signaling is critical for maintaining spine structure, and disruption of store-operated calcium entry promotes mushroom spine destabilization and increases susceptibility to amyloid toxicity (Popugaeva et al., 2015; Zhang et al., 2015). In the 5xFAD mouse model, rapid amyloid accumulation is accompanied by early synaptic dysfunction and deficits in learning and memory (Oakley et al., 2006; Kimura and Ohno, 2009). In addition to cognitive impairment, 5xFAD mice also exhibit alterations in social behavior, anxiety-like responses, and depressive-like phenotypes, reflecting dysfunction of hippocampal and limbic circuits (Jawhar et al., 2012; Kosel et al., 2020). Similar non-cognitive symptoms, including anxiety, depression, and social withdrawal, are clinically relevant features of AD (Zhao et al., 2016; Banning et al., 2019).

Systemic inflammation and peripheral infections are increasingly recognized as modifiers of AD pathology. Peripheral immune activation can induce neuroinflammatory responses through cytokine signaling and glial activation, potentially accelerating cognitive decline and neurodegenerative processes (Perry et al., 2007; Cunningham, 2013). Viral infections are of particular interest because they can produce sustained inflammatory responses and alter neuronal function Influenza A virus, although primarily respiratory, has also been associated with neurological complications and long-term effects on brain function (Bohmwald et al., 2018).

Experimental studies have shown that influenza infection can induce persistent neuroinflammation and alter hippocampal function even in the absence of direct viral invasion of the brain. Influenza-associated microglial activation, changes in neuronal morphology, impaired synaptic plasticity, and spatial-memory deficits have been reported in mice (Jurgens et al., 2012; Hosseini et al., 2018). These infection-induced effects may converge on calcium-dependent pathways regulating synaptic stability, thereby enhancing vulnerability to neurodegeneration (Bezprozvanny, 2026). Infection-induced inflammation may also affect non-cognitive behavior, as pro-inflammatory cytokine signaling has been associated with anxiety-, depressive-, and social-behavior alterations (Dantzer et al., 2008; Yirmiya et al., 2015). Thus, inflammatory pathways activated by viral infection may overlap with mechanisms already dysregulated in AD.

Despite accumulating evidence linking systemic infection to neurodegeneration, it remains unclear how prior viral exposure modifies behavioral dysfunction, synaptic pathology, and neuroinflammatory responses in established AD. In particular, the relationship between infection-induced behavioral alterations and changes in synaptic and glial pathology remains poorly defined. Here, we combined the 5xFAD mouse model with prior influenza A (H1N1)pdm infection to determine whether viral exposure modifies cognitive and non-cognitive behavior, hippocampal synaptic structure and plasticity, and neuroinflammatory responses. We hypothesized that prior H1N1pdm infection would modify AD-related phenotypes through interactions between systemic inflammatory stress and pre-existing neurodegenerative pathology.

## 2 Results

### 2.1 Development of 5xFAD viral infection model

In the present study, we used the 5xFAD transgenic mouse model of Alzheimer’s disease combined with prior infection by influenza A (H1N1)pdm to investigate the effects of viral exposure on behavior, cognition, and synaptic structure. Homozygous mutations are associated with early death of 5xFAD mice. Therefore, we crossbreeded male 5xFAD and female M-line mice to create heterozygous 5xFAD-M line that is referred as 5xFAD. Newborn mice were genotyped about 1 month after birth and divided into 5xFAD and M-line (WT) mice. At 2 months of age, mice were intranasally infected with H1N1pdm virus in a sublethal dose (Figure 1). Viral infectious dose was determined in a preliminary experiment (Supplementary Figure S1). The dose that causes the death of a half of infected animals was the same for 5xFAD and WT mice. Influenza infection in this dose (4.5 lg TCID_50_/mL) resulted in significant morbidity and mortality in both WT and 5xFAD mice (Supplementary Figure S2).

**Figure 1:**
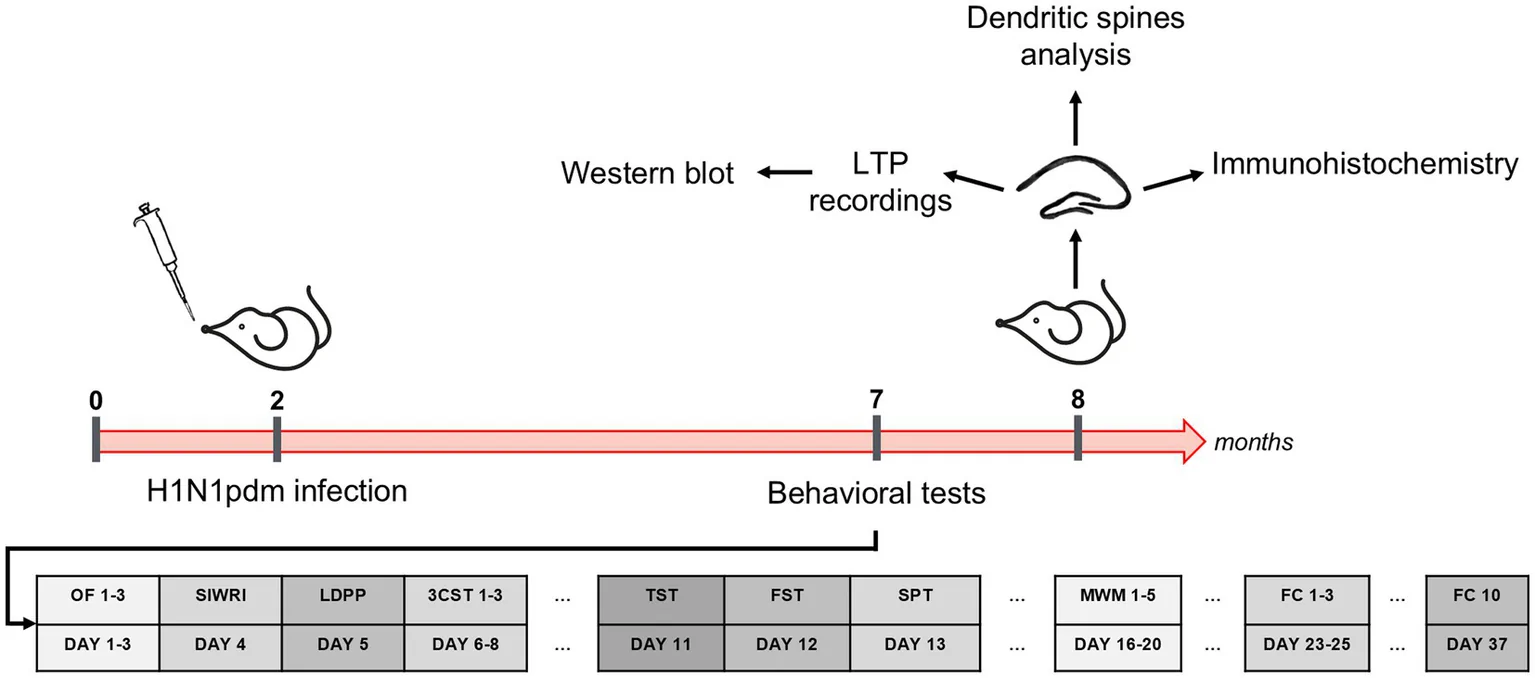
A graphical time scheme of the experiments on the 5xFAD + H1N1pdm mice. OF S1, open field stage 1; SIWRI, social interaction with resident intruder; LDPP, light-dark place preference; 3CST1-3, three-chamber social test day 1–3; TST, tail suspension test; FST, forced swimming test; SPT, sucrose preference test; MWM1, Morris water maze day 1; FC1, fear conditioning day 1; LTP recordings: recording long-term potentiation. Dots represent the rest days.

### 2.2 Reduced social interaction and novel object interest in 5xFAD mice following H1N1pdm infection

To evaluate anxiety-like behavior and exploratory activity, mice were subjected to the open field (OF) test with a novel non-social object (a black metal mesh cylinder) placed in the center of the arena. Mice of the 5xFAD + H1N1pdm group spent significantly less time exploring the object compared to WT mice exposed to the virus (WT + H1N1pdm: 107.1 ± 9.89 s; 5xFAD + H1N1pdm: 59.74 ± 16.92 s; Figure 2A). Similarly, the number of approaches to the object was reduced in 5xFAD + H1N1pdm mice (WT + H1N1pdm: 58.67 ± 3.58; 5xFAD: 61.08 ± 4.13; 5xFAD + H1N1pdm: 40.13 ± 5.41). On the third day of the OF test, a social stimulus (an unfamiliar mouse of the same sex) was placed under the cylinder. Under these conditions, 5xFAD + H1N1pdm mice exhibited altered exploration patterns, characterized by reduced investigation of the centrally located social stimulus (WT + H1N1pdm: 44.18 ± 6.07 s; 5xFAD: 38.84 ± 6.18 s; 5xFAD + H1N1pdm: 77.61 ± 9.51 s). However, interpretation of OF results is limited due to the central placement of stimuli, as reduced center exploration may reflect either increased anxiety or decreased object-directed behavior.

**Figure 2:**
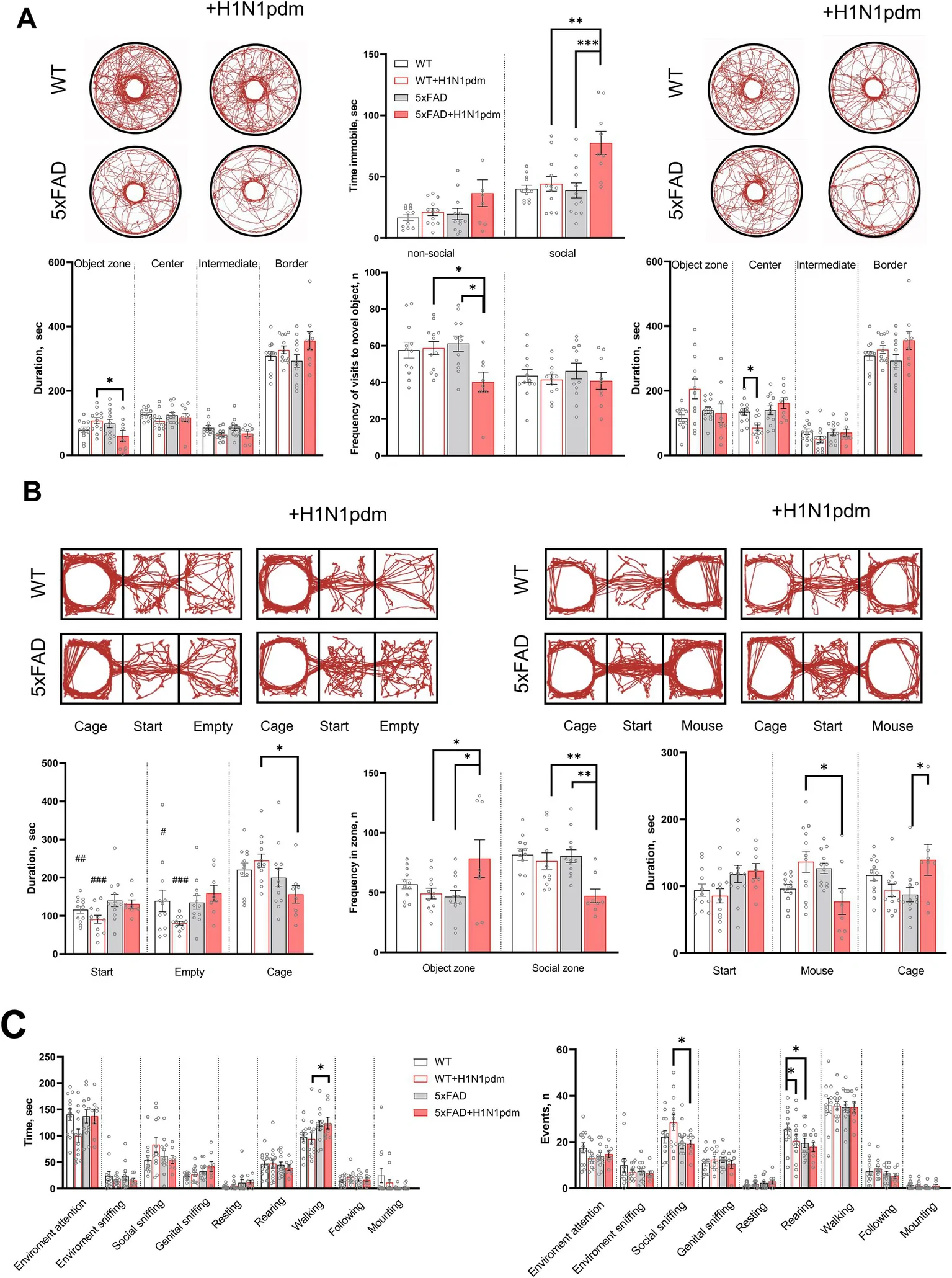
Reduced social interaction and novel object interest in 5xFAD mice following H1N1pdm infection. **(A)** Open field (OF). Representative tracks of WT, WT + H1N1pdm, 5xFAD, and 5xFAD + H1N1pdm mice during exploration in the OF test with a non-social object (day 2, left) and a social object (day 3, right). Immobility time, time spent in different zones, and the number of approaches to the object are shown. **(B)** Three-chamber social test (3CST). Top: representative trajectories on day 2 (object vs. empty chamber, left) and day 3 (object vs. mouse, right). Bottom: time spent in each chamber and the number of visits to the objects. **(C)** Social interaction test (SIWRI): duration and frequency of behavioral events, including social investigation, sniffing, locomotion, and exploratory activity during interaction with an unfamiliar mouse. Data are presented as mean ± SEM. Group sizes: WT, *n* = 12; WT + H1N1pdm, *n* = 12; 5xFAD, *n* = 11; 5xFAD + H1N1pdm, *n* = 8. Asterisks indicate comparisons between experimental groups and were analyzed using two-way ANOVA with genotype and infection status as independent factors, followed by Tukey’s multiple-comparisons test where appropriate (*p < 0.05, **p < 0.01, ***p < 0.001). Hash symbols indicate comparisons between different zones within the same experimental group and were analyzed using one-way ANOVA followed by Tukey’s multiple-comparisons test (^#^*p* < 0.05, ^##^*p* < 0.01, ^###^*p* < 0.001).

However, the circular OF paradigm has important limitations. Both non-social and social stimuli were positioned in the center of the arena, and mice may have been less likely to explore the center due to increased anxiety. Therefore, reduced center exploration cannot be unambiguously interpreted as decreased object interest, as it may instead reflect anxiety-related avoidance of the central zone. Additionally, during the social phase of the OF test, it remains unclear whether the observed behavior reflects true social interaction deficits or differences in habituation to the arena. To further assess social preference and decision-making, mice were tested in the three-chamber social test (3CST) (Figure 2B). During the object exploration phase, WT and WT + H1N1pdm mice showed comparable behavior, spending more time in the chamber containing the novel object compared to empty chambers (WT: 220.9 ± 17.41 s; WT + H1N1pdm: 244.6 ± 17.50 s). In contrast, 5xFAD + H1N1pdm mice demonstrated reduced interest in the object (156.2 ± 23.00 s vs. WT + H1N1pdm). During the social interaction phase, 5xFAD + H1N1pdm mice exhibited impaired social preference, spending less time in the chamber with the unfamiliar mouse compared to WT + H1N1pdm controls (136.8 ± 15.76 s vs. 77.05 ± 19.48 s). This was accompanied by a reduced number of entries into the social chamber (WT + H1N1pdm: 76.50 ± 6.76; 5xFAD: 80.67 ± 5.33; 5xFAD + H1N1pdm: 47.25 ± 5.80). Conversely, time spent in the chamber with the non-social object increased (5xFAD: 87.45 ± 10.89 s; 5xFAD + H1N1pdm: 139.4 ± 139.4 s), along with the number of entries into this zone (WT + H1N1pdm: 49.17 ± 4.45; 5xFAD: 46.50 ± 5.19; 5xFAD + H1N1pdm: 78.50 ± 15.68), indicating a shift in preference from social to non-social stimuli.

In the social interaction test (SIWRI) (Figure 2C), both WT + H1N1pdm and 5xFAD mice exhibited reduced exploratory activity, as indicated by decreased rearing behavior (WT: 28.40 ± 1.84; WT + H1N1pdm: 20.42 ± 2.87; 5xFAD: 19.58 ± 2.01). In line with the findings from the previous test, 5xFAD + H1N1pdm mice showed reduced social interaction, characterized by a lower number of social sniffing events (WT + H1N1pdm: 29.91 ± 3.39; 5xFAD + H1N1pdm: 19.13 ± 1.75) and increased walking duration (WT + H1N1pdm: 85.51 ± 7.67; 5xFAD + H1N1pdm: 123.9 ± 11.19). Overall, 5xFAD mice that recovered from H1N1pdm infection (5xFAD + H1N1pdm) exhibited pronounced alterations in social behavior, particularly in the 3CST, while reduced social interest was also evident in 5xFAD mice in the SIWRI paradigm.

### 2.3 Anxiolytic-like behavior in 5xFAD mice and increased depressive-like phenotype following H1N1pdm infection

To assess depressive-like behavior, mice from all experimental groups (WT, WT + H1N1pdm, 5xFAD, and 5xFAD + H1N1pdm) were subjected to the tail suspension test (TST), forced swim test (FST) and sucrose preference test (SPT) (Figure 3A). In the TST, immobility time was analyzed across the entire 6-min test as well as in a time-resolved manner, assessed for each minute interval (from 1 to 6 min). No significant differences in total immobility time were observed between the groups. However, time-resolved analysis revealed that 5xFAD + H1N1pdm mice exhibited a significant increase in immobility during the first minute of the test compared to both WT + H1N1pdm and 5xFAD mice, indicating an early onset of depressive-like behavior (WT + H1N1pdm: 5.17 ± 1.46; 5xFAD: 3.82 ± 1.77; 5xFAD + H1N1pdm: 6.13 ± 2.72). In contrast, in the forced swim test (FST), no significant differences were detected between groups either in total immobility time or in immobility assessed across individual time intervals, suggesting the absence of robust depressive-like alterations in this paradigm (Figure 3B). In addition, no evidence of anhedonia was observed in the SPT. During this test, mice were given a choice between water and a 2% sucrose solution. Both sucrose preference and total fluid intake were comparable across all groups, including 5xFAD + H1N1pdm mice, indicating preserved reward sensitivity (Figure 3C).

**Figure 3:**
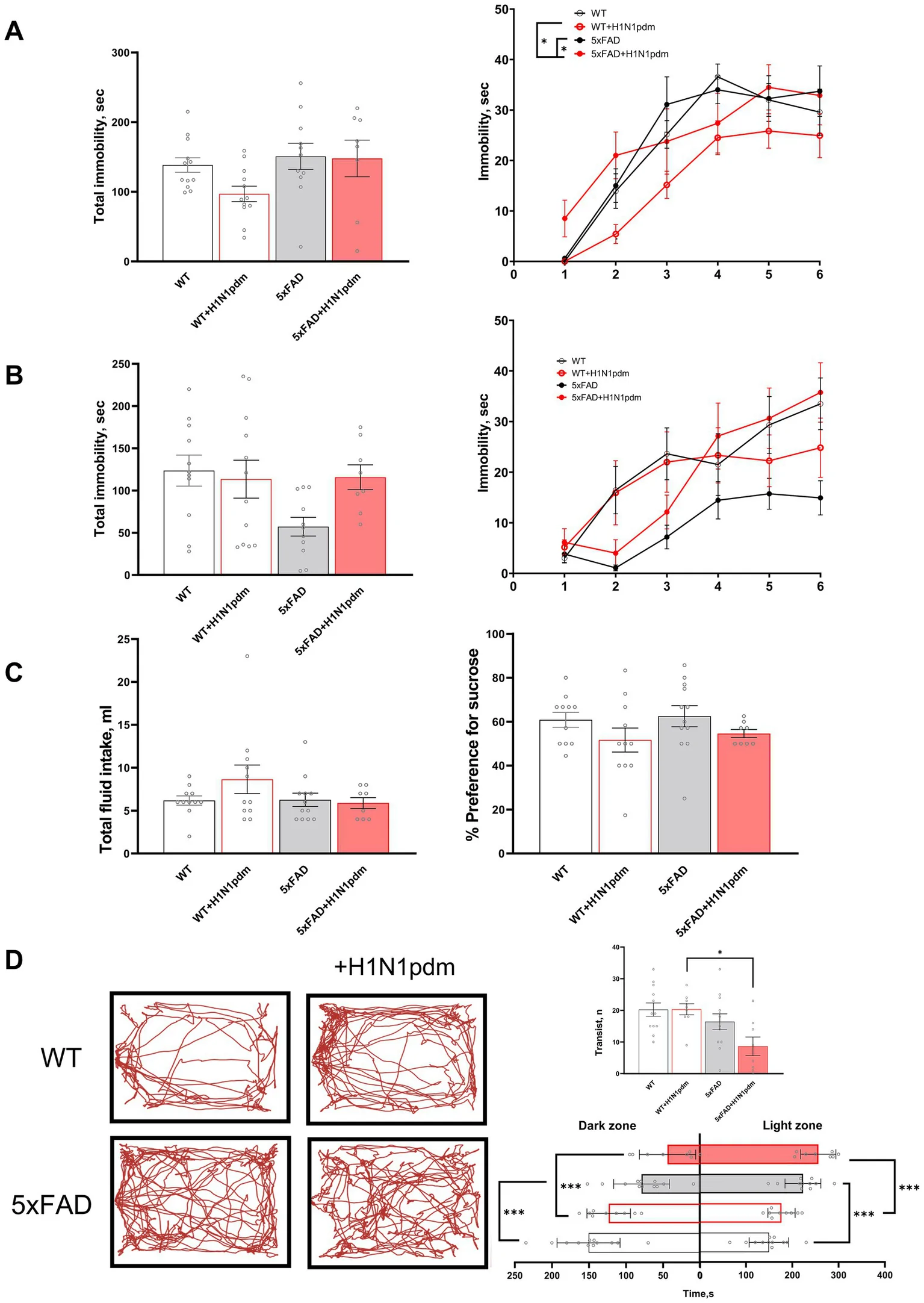
Anxiolytic-like behavior in 5xFAD mice and increased depressive-like phenotype following H1N1pdm infection. **(A)** Tail suspension test (TST): total immobility time and immobility dynamics across 1-min intervals. **(B)** Forced swim test (FST): total immobility time and immobility dynamics across 1-min intervals. **(C)** Sucrose preference test (SPT): total fluid intake and sucrose preference (%). **(D)** Light–dark place preference test (LDPP): representative tracks in the light compartment, number of transitions between compartments, and time spent in light and dark zones. Data are presented as mean ± SEM. Group sizes: WT, *n* = 12; WT + H1N1pdm, *n* = 12; 5xFAD, *n* = 11; 5xFAD + H1N1pdm, *n* = 8. Comparisons between experimental groups were performed using two-way ANOVA with genotype (WT vs. 5xFAD) and infection status (control vs. H1N1pdm) as independent factors, followed by Tukey’s post-hoc multiple-comparisons test where appropriate (*p < 0.05, ***p < 0.001).

To evaluate anxiety-like behavior, mice were tested in the light–dark place preference (LDPP) test (Figure 3D). In this test, 5xFAD + H1N1pdm mice displayed an anxiolytic-like phenotype, as evidenced by increased time spent in the light compartment (WT + H1N1pdm: 176.7 ± 9.80 s; 5xFAD + H1N1pdm: 256.10 ± 13.44 s) and decreased time spent in the dark compartment compared to WT + H1N1pdm mice (WT + H1N1pdm: 123.3 ± 9.80 s; 5xFAD + H1N1pdm: 43.88 ± 13.44 s). A similar tendency toward increased time in the light compartment was also observed in 5xFAD mice compared to WT (WT: Light: 149.6 ± 12.28 s and Dark: 150.40 ± 12.28 s; 5xFAD: Light: 223.00 ± 11.67 s and Dark: 79.00 ± 10.84 s). Additionally, the number of transitions between compartments was reduced in 5xFAD + H1N1pdm mice, suggesting altered exploratory behavior (WT + H1N1pdm: 20.33 ± 1.76; 5xFAD + H1N1pdm: 8.63 ± 2.95). Taken together, these results indicate that 5xFAD mice following H1N1pdm infection exhibit increased depressive-like behavior in the TST, without signs of anhedonia in the FST and SPT, and display an anxiolytic-like phenotype in the LDPP test.

### 2.4 Reduced contextual memory in 5xFAD mice and impaired spatial memory following H1N1pdm infection

To assess cognitive function, mice were subjected to the Morris water maze (MWM) and fear conditioning (FC) tests, which are closely associated with hippocampus-dependent memory. In the MWM test (Figure 4A), all groups demonstrated learning over the training period, as indicated by a progressive decrease in latency to locate the hidden platform (WT: Day 1: 77.63 ± 4.42 s and Day 4: 50.31 ± 5.98 s, *p* < 0.01; WT + H1N1pdm: Day 1: 71.00 ± 4.84 s and Day 4: 53.45 ± 4.46 s, *p* < 0.05; 5xFAD: Day 1: 77.36 ± 4.57 s and Day 4: 59.86 ± 5.31 s, *p* < 0.05; 5xFAD + H1N1pdm: Day 1: 75.59 ± 4.45 s and Day 4: 51.00 ± 4.79 s, *p* < 0.01) (Figure 4). During the probe trial, time spent in the platform zone was significantly reduced in WT + H1N1pdm and 5xFAD mice compared to WT controls (WT: 2.20 ± 0.87; WT + H1N1pdm: 0.47 ± 0.12; 5xFAD: 0.41 ± 0.13) (Figure 4). To further examine the relationship between spatial memory performance and hippocampal synaptic alterations, individual MWM performance was subsequently correlated with dendritic spine parameters obtained from the same animals. Consistently, the number of platform crossings was significantly decreased in 5xFAD + H1N1pdm mice compared to 5xFAD mice (5xFAD: 1.55 ± 0.28; 5xFAD + H1N1pdm: 0.42 ± 0.20) (Figure 4). Importantly, no differences in swimming speed were observed between groups, indicating that the observed deficits were not due to motor impairments.

**Figure 4:**
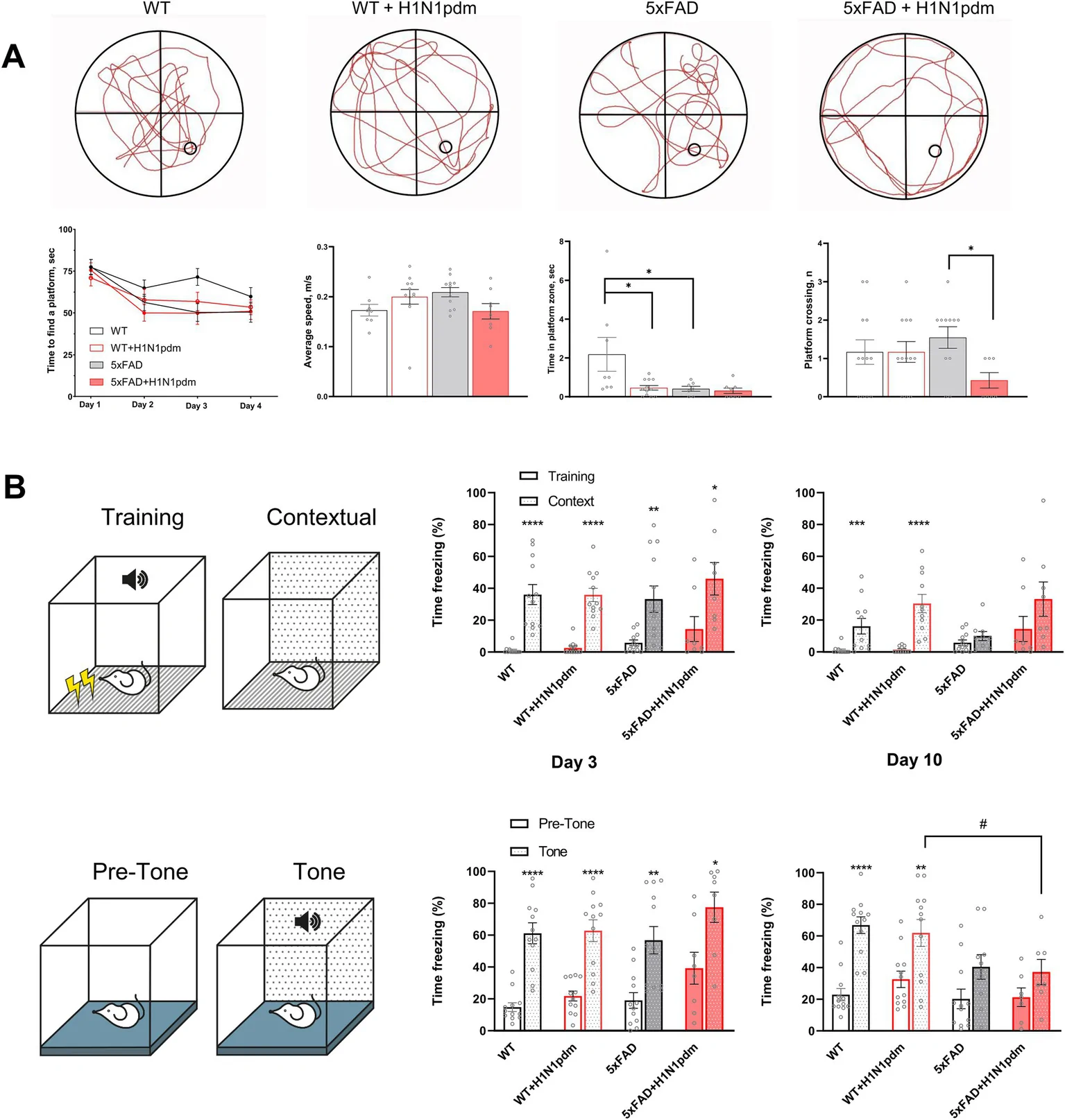
Reduced contextual memory in 5xFAD mice and impaired spatial memory following H1N1pdm infection. **(A)** Morris water maze. Top: representative swimming trajectories of each experimental group during the probe trial. Bottom: latency to reach the platform during training, average swimming speed, time spent in the target quadrant during the probe test, and number of platform location crossings. **(B)** Fear conditioning (FC). Left: schematic of the experimental design. Top: total contextual freezing (%) measured on Days 3 and 10. Bottom: total cued freezing (%) measured on Days 3 and 10. Data are presented as mean ± SEM. Group sizes: WT, *n* = 12; WT + H1N1pdm, *n* = 12; 5xFAD, *n* = 11; 5xFAD + H1N1pdm, *n* = 8. Asterisks indicate within-group comparisons between experimental conditions (*p < 0.05, **p < 0.01, ***p < 0.001, ****p < 0.0001; Mann–Whitney test), whereas hash symbols indicate comparisons between different experimental groups under the same condition or time point (^#^*p* < 0.05; Kruskal–Wallis test followed by Dunn’s multiple-comparisons test).

To further validate these findings, associative learning and memory were assessed using the FC test (Figure 4B). On Day 3, all groups showed a significant increase in freezing behavior compared to baseline during both contextual and cued phases, indicating successful acquisition of fear memory (contextual: WT: 1.02 ± 0.73% vs. 36.08 ± 6.29%; WT + H1N1pdm: 2.52 ± 1.24% vs. 35.85 ± 4.23%; 5xFAD: 5.75 ± 1.76% vs. 33.20 ± 8.36%; 5xFAD + H1N1pdm: 14.41 ± 7.84% vs. 45.94 ± 10.17%; cued: WT: 14.81 ± 2.72% vs. 61.16 ± 6.54%; WT + H1N1pdm: 21.76 ± 3.12% vs. 62.78 ± 6.85%; 5xFAD: 19.01 ± 4.87% vs. 56.80 ± 8.61%; 5xFAD + H1N1pdm: 39.25 ± 10.06% vs. 77.45 ± 9.52%), and is consistent with previously published studies (Gerasimov et al., 2025; Zernov et al., 2025). However, on Day 10, 5xFAD and 5xFAD + H1N1pdm mice did not show significant differences between baseline and contextual freezing, indicating impaired long-term contextual memory. In contrast, WT and WT + H1N1pdm mice maintained significant differences between training and testing phases (WT: 1.02 ± 0.73% vs. 16.14 ± 4.94%; WT + H1N1pdm: 2.52 ± 1.24% vs. 30.41 ± 5.71%). A similar pattern was observed in the cued test, where WT and WT + H1N1pdm mice retained robust freezing responses on Day 10 (WT: 22.89 ± 3.89% vs. 66.88 ± 5.24%; WT + H1N1pdm: 32.59 ± 5.17% vs. 61.95 ± 8.54%). In contrast, 5xFAD + H1N1pdm mice exhibited a significant reduction in cued freezing compared to WT + H1N1pdm mice (37.17 ± 8.00% vs. 61.95 ± 8.54%). Taken together, these results demonstrate that 5xFAD mice exhibit deficits in both spatial and contextual memory, and that prior H1N1pdm infection exacerbates these impairments, particularly at later stages of memory consolidation.

### 2.5 H1N1pdm infection alters hippocampal synaptic plasticity and dendritic spine morphology

We conducted experiments to assess long-term potentiation in a control group of wild-type (WT) mice and mice with Alzheimer’s disease (5xFAD), as well as in experimental groups following exposure to the H1N1pdm influenza virus (Figure 5A). After HFS, the fEPSP slope increased in all groups, followed by a gradual decrease. The slope percentage values normalized to baseline (LTP, last 10 min after HFS) were significantly lower in 5xFAD mice than in WT mice in the control group (WT: 161.86 ± 7.63; 5xFAD: 131.93 ± 3.55), which is consistent with previously reported data in the literature (Rakovskaya et al., 2023). At the same time, significant differences in the magnitude of long-term potentiation were observed in the experimental group between wild-type mice and 5xFAD mice following exposure to the H1N1pdm virus (WT + H1N1pdm: 160.81 ± 6.43; 5xFAD + H1N1pdm: 138.22 ± 1.93). However, no statistically significant differences were observed between the control group of WT mice and WT mice after influenza virus exposure, as well as no differences between 5xFAD mice before and after H1N1pdm virus exposure.

**Figure 5:**
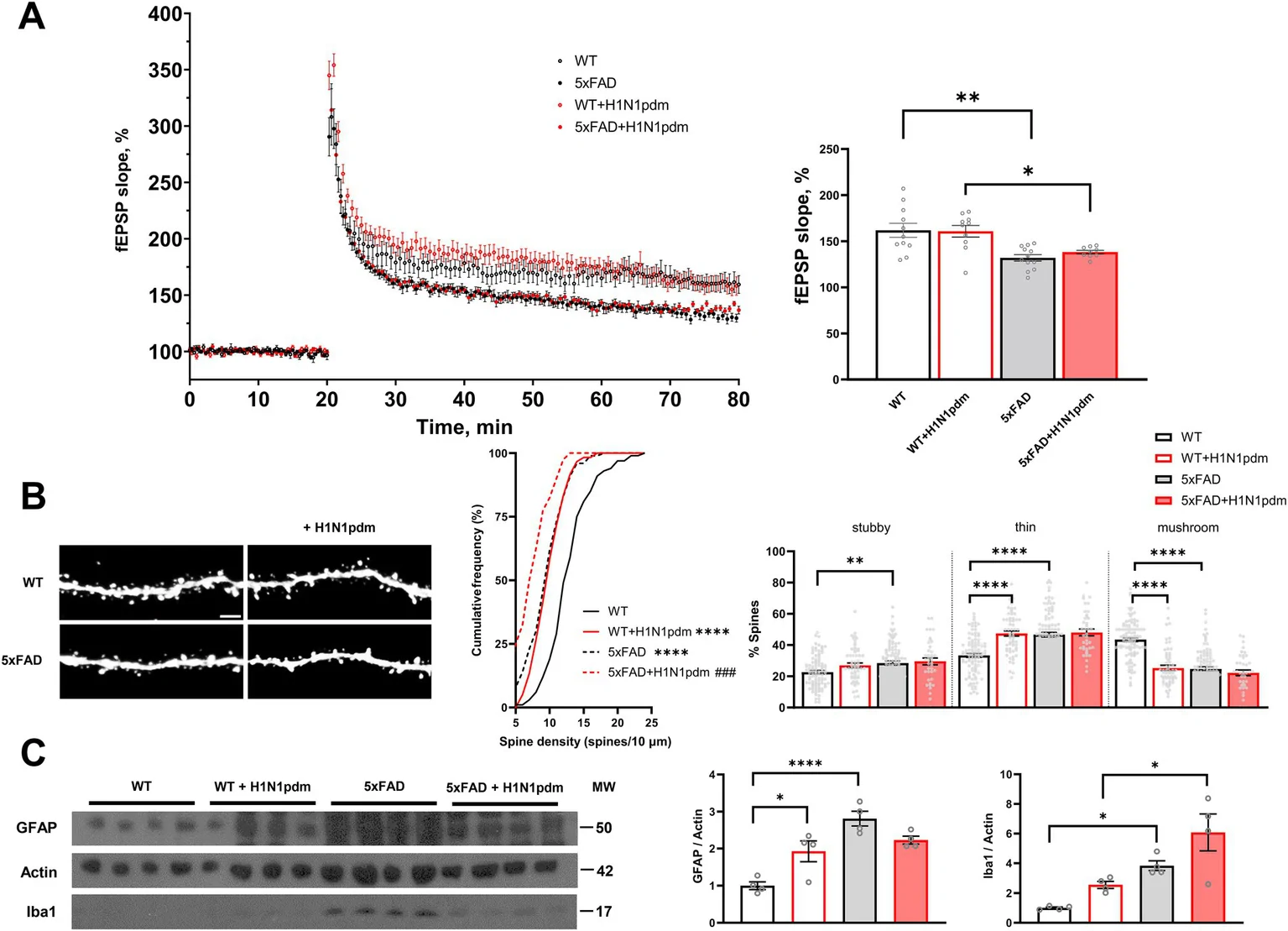
H1N1pdm infection alters hippocampal synaptic plasticity, dendritic spine morphology, and glial marker expression. **(A)** Left panel: The plot shows the combined graphs of changes in the normalized fEPSP slope (%) over the recording period for all groups of mice. Data are presented as the mean ± SEM. Right panel: Normalized fEPSP slope (%) for long-term potentiation (last 10 min after high-frequency stimulation). A significant reduction in LTP was observed in 5xFAD mice compared to WT mice. Furthermore, LTP levels were significantly lower in 5xFAD mice compared to WT mice following exposure to the H1N1pdm virus. Groups (*n* = LTP recordings): WT: *n* = 11; 5xFAD: *n* = 12; WT + H1N1pdm: *n* = 10; 5xFAD + H1N1pdm: *n* = 9. Statistical analysis was performed using two-way ANOVA with Tukey’s post-hoc test: *p < 0.05, **p < 0.01. **(B)** Left panel: Representative images of dendritic spines from hippocampal neurons in WT and 5xFAD mice with or without H1N1pdm infection. Scale bar: 2 μm. Right panel: Cumulative frequency distribution of dendritic spine density (spines/10 μm), demonstrating a leftward shift in 5xFAD and infected groups. For dendritic spine analysis, 20, 12, 20, and 8 hippocampal sections and 100, 60, 100, and 40 dendritic segments were analyzed in the WT, WT + H1N1pdm, 5xFAD, and 5xFAD + H1N1pdm groups, respectively. Quantification of dendritic spine subtype proportions (stubby, thin and mushroom) (right). Statistical analysis was performed using two-way ANOVA with Tukey’s post-hoc test; **p < 0.01, ****p < 0.0001 vs. WT; ^#^^#^^#^*p* < 0.001 vs. 5xFAD. **(C)** Left panel: Western blot analysis of GFAP and Iba1 protein expression in the hippocampus of WT and 5xFAD mice with or without H1N1pdm infection. Representative immunoblots (left), with β-actin used as a loading control. Right panel: Quantification of GFAP and Iba1 expression levels normalized to β-actin. Western blot analysis was performed using hippocampal samples from *n* = 4 mice per group. Data are presented as mean ± SEM; Statistical analysis was performed using two-way ANOVA followed by Tukey’s post-hoc test; *p < 0.05, ***p < 0.001.

Quantitative analysis of dendritic spines in hippocampal neurons revealed significant changes in both overall spine density and morphological composition following H1N1pdm infection (Figure 5B). In 5xFAD mice, a statistically significant reduction in dendritic spine density was observed compared to the wild-type (WT) control group (WT: 13.14 ± 0.33; 5xFAD: 9.74 ± 0.29). This finding is consistent with numerous reports describing progressive dendritic spine loss and synaptic dysfunction in Alzheimer’s disease (Scheff et al., 2006). Importantly, prior H1N1pdm influenza infection resulted in a decrease in dendritic spine density in WT + H1N1pdm mice compared to WT controls (WT: 13.14 ± 0.33; WT + H1N1pdm: 10.15 ± 0.31). Consequently, viral infection acts as an independent negative factor reducing synaptic density but does not further exacerbate the already existing deficit in the Alzheimer’s disease model. In addition to quantitative changes, the distribution of spines across three main morphological types (thin, mushroom and stubby) was analyzed. Compared to WT mice, 5xFAD mice exhibited a marked shift in spine profile: the proportion of mushroom spines was significantly decreased (WT: 43.53 ± 1.21%; 5xFAD: 24.82 ± 1.23%), whereas the proportion of thin spines was increased (WT: 33.41 ± 1.21%; 5xFAD: 46.72 ± 2.16%). The proportion of stubby spines was increased in 5xFAD mice compared to WT (WT: 22.69 ± 1.03%; 5xFAD: 28.51 ± 1.29%), further indicating a shift toward less mature spine phenotypes. Importantly, individual time spent in the target platform zone in the Morris water maze was positively correlated with the proportion of mature mushroom spines measured in the same animals (Spearman *r*_s_ = 0.556, *p* = 0.0485, *n* = 13). Thus, better spatial memory performance was associated with a higher proportion of mature dendritic spines. This is a well-established hallmark of synaptic instability and impaired plasticity, widely reported in transgenic models of Alzheimer’s disease (Shankar et al., 2007). H1N1pdm influenza infection induced similar morphological alterations. In the WT + H1N1pdm group, a significant increase in the proportion of thin spines (WT: 33.41 ± 1.21%; WT + H1N1pdm: 47.52 ± 1.71%) and a decrease in mushroom spines (WT: 43.53 ± 1.21%; WT + H1N1pdm: 25.39 ± 1.67%) were also observed compared to WT controls. These findings indicate that viral infection alone can induce a state of synaptic immaturity and instability. Neuroinflammation, induced both by amyloid pathology in 5xFAD mice and by systemic infection, may disrupt cytoskeletal organization and signaling pathways required for maintaining mature spine morphology (Hong et al., 2016).

Neuroinflammation is a key response to viral infection and is characterized by increased levels of pro-inflammatory cytokines, activation of microglia, and astrocytic reactivity, which may ultimately contribute to neuronal damage (Woodburn et al., 2021). Microglia, the resident immune cells of the brain, play a central role in this process, and their activation is commonly assessed using the marker Iba1. In parallel, astrocytes contribute to both pro-inflammatory and repair processes, with reactive astrogliosis typically associated with increased expression of GFAP (Colombo and Farina, 2016). Western blot analysis revealed significant changes in GFAP and Iba1 protein expression in the hippocampus following the influence of H1N1pdm infection (Figure 5C). In 5xFAD mice, a significant increase in GFAP expression was observed compared to WT controls (WT: 1.00 ± 0.10; 5xFAD: 2.81 ± 0.20), indicating enhanced astrocyte activation in the transgenic model (Hong et al., 2016). A similar increase was detected following H1N1pdm infection, where GFAP levels were significantly elevated in WT + H1N1pdm mice compared to WT (WT: 1.00 ± 0.10; WT + H1N1pdm: 1.93 ± 0.28). Analysis of Iba1 expression showed a comparable pattern. In 5xFAD mice, Iba1 levels were significantly increased relative to WT controls (WT: 1.00 ± 0.06; 5xFAD: 3.84 ± 0.33), reflecting microglial activation. Following H1N1pdm infection, Iba1 expression was elevated in 5xFAD + H1N1pdm mice compared to infected WT animals (WT + H1N1pdm: 2.55 ± 0.24; 5xFAD + H1N1pdm: 6.08 ± 1.25). Overall, these results indicate that both amyloid pathology and viral infection contribute to enhanced astrocytic and microglial activation in the hippocampus, with the strongest effects observed in 5xFAD mice following H1N1pdm infection.

### 2.6 H1N1pdm infection enhances glial reactivity and induces distinct astrocytic and microglial morphological remodeling in the hippocampus

Quantitative immunohistochemical analysis revealed significant changes in β-amyloid deposition and glial activation in the hippocampus following H1N1pdm infection (Figures 6A–C). In 5xFAD mice, a significant increase in the percentage area occupied by β-amyloid plaques relative to the total hippocampal area was observed compared to WT controls (WT: 0.79 ± 0.08%; 5xFAD: 3.0 ± 0.31%), as well as an increase in the average plaque size (WT: 137.00 ± 4.48 µm^2^; 5xFAD: 182.90 ± 11.34 µm^2^), indicating substantially higher amyloid deposition in the transgenic model. These findings confirm the presence of amyloid pathology in the hippocampus, a key brain region involved in learning and memory, and a central hallmark of Alzheimer’s disease (Oakley et al., 2006). Following H1N1pdm influenza infection, amyloid burden remained elevated in 5xFAD mice compared to infected WT animals. This was reflected by an increase in both the plaque area percentage (WT + H1N1pdm: 0.67 ± 0.12%; 5xFAD + H1N1pdm: 3.51 ± 0.25%) and the average plaque size (WT + H1N1pdm: 144.0 ± 7.06 µm^2^; 5xFAD + H1N1pdm: 192.90 ± 8.90 µm^2^). Quantitative analysis of glial fibrillary acidic protein (GFAP), a marker of reactive astrogliosis, revealed increased astrocyte activation in the hippocampus of 5xFAD mice compared to WT controls. This is consistent with previous reports of neuroinflammation associated with amyloid pathology in transgenic models of Alzheimer’s disease (Hong et al., 2016). This was reflected by both an increase in the average area of GFAP-positive cells (WT: 144.10 ± 4.25 µm^2^; 5xFAD: 177.50 ± 4.29 µm^2^) and the percentage area they occupied (WT: 5.73 ± 0.16%; 5xFAD: 7.75 ± 0.21%). A similar pattern was observed following H1N1pdm infection, where both parameters remained significantly elevated in 5xFAD + H1N1pdm mice compared to WT + H1N1pdm animals (average area: WT + H1N1pdm: 140.80 ± 4.13 µm^2^; 5xFAD + H1N1pdm: 180.10 ± 13.42 µm^2^; percentage area: WT + H1N1pdm: 5.71 ± 0.23%; 5xFAD + H1N1pdm: 7.63 ± 0.17%). These changes suggest astrocyte hypertrophy consistent with a reactive phenotype under neuroinflammatory conditions. To further characterize astrocytic reactivity at the cellular level, high-magnification morphological analysis of individual GFAP-positive astrocytes was performed (Figure 6B). Astrocytes in 5xFAD mice showed an expansion of the cellular arbor and increased morphological complexity, reflected by increases in the number of branch points (WT: 97.2 ± 9.475; 5xFAD: 168.6 ± 15.78), convex hull size (WT: 619.7 ± 63.97 µm^2^; 5xFAD: 1139 ± 72 µm^2^), Sholl sum (WT: 3191 ± 305; 5xFAD: 5476 ± 393.6), and surface area (WT: 193.6 ± 21.38 µm^2^; 5xFAD: 395.9 ± 39.62 µm^2^) compared with WT mice. H1N1pdm infection did not result in a uniform additional increase in these parameters in the 5xFAD background; however, branch length (WT + H1N1pdm: 1.383 ± 0.024 µm; 5xFAD: 1.513 ± 0.047 µm; 5xFAD + H1N1pdm: 1.793 ± 0.062 µm) was significantly increased in 5xFAD + H1N1pdm mice compared with both 5xFAD and WT + H1N1pdm animals. These findings indicate pronounced astrocytic morphological remodeling associated with 5xFAD pathology, whereas the additional effect of H1N1pdm infection was selective rather than uniformly additive.

**Figure 6:**
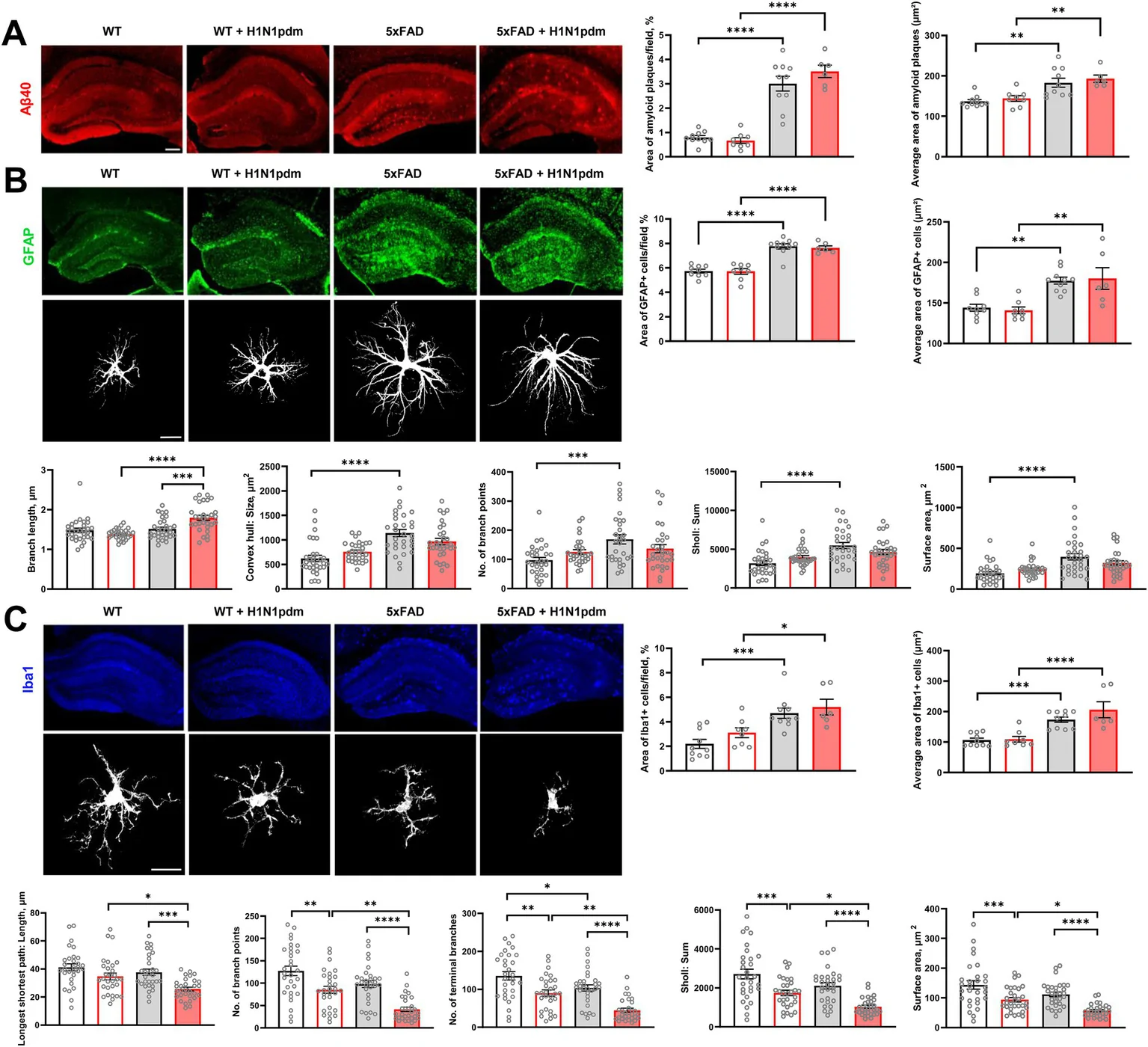
H1N1pdm infection is associated with amyloid pathology and morphological remodeling of astrocytes and microglia in the hippocampus. **(A)** Representative immunofluorescence images of hippocampal sections stained for Aβ40 in WT, WT + H1N1pdm, 5xFAD, and 5xFAD + H1N1pdm mice. Quantitative analysis shows the percentage area occupied by amyloid plaques and the average plaque area. **(B)** Representative immunofluorescence images of GFAP-positive astrocytes and high-magnification examples of individual astrocytes used for morphological analysis. Quantitative analysis includes the percentage area occupied by GFAP-positive cells, average GFAP-positive cell area, branch length, convex hull size, number of branch points, Sholl sum, and surface area. **(C)** Representative immunofluorescence images of Iba1-positive microglia and high-magnification examples of individual microglial cells used for morphological analysis. Quantitative analysis includes the percentage area occupied by Iba1-positive cells, average Iba1-positive cell area, longest shortest path length, number of branch points, number of terminal branches, Sholl sum, and surface area. Immunohistochemical analysis included 10, 8, 10, and 6 hippocampal sections obtained from 5, 4, 5, and 3 mice in the WT, WT + H1N1pdm, 5xFAD, and 5xFAD + H1N1pdm groups. For Sholl analysis, 30 cells per group were analyzed. Data are presented as mean ± SEM. Statistical analysis was performed using two-way ANOVA with genotype and infection status as independent factors, followed by Tukey’s post-hoc multiple-comparisons test where appropriate. *p < 0.05, **p < 0.01, ***p < 0.001, ****p < 0.0001.

Immunohistochemical analysis using the microglial marker Iba1 revealed activation of resident immune cells in the hippocampus of 5xFAD mice compared to WT controls. Both the percentage area occupied by Iba1-positive cells (WT: 2.20 ± 0.37%; 5xFAD: 4.70 ± 0.42%) and their average cell area (WT: 106.60 ± 6.61 µm^2^; 5xFAD: 173.40 ± 8.84 µm^2^) were increased, indicating a transition to a reactive microglial phenotype characteristic of neuroinflammation (Hong et al., 2016). This effect was further enhanced following H1N1pdm infection, with both the percentage area (WT + H1N1pdm: 3.11 ± 0.39%; 5xFAD + H1N1pdm: 5.20 ± 0.64%) and average cell area (WT + H1N1pdm: 109.40 ± 9.38 µm^2^; 5xFAD + H1N1pdm: 205.90 ± 26.30 µm^2^) being significantly higher in 5xFAD + H1N1pdm mice compared to infected WT animals. Detailed morphological analysis of individual Iba1-positive microglia revealed marked remodeling of the microglial arbor following H1N1pdm infection (Figure 6C). Infection was associated with reduced process complexity, including decreases in the number of branch points (WT: 127.6 ± 10.5; WT + H1N1pdm: 85.07 ± 7.862; 5xFAD: 97.9 ± 7.867; 5xFAD + H1N1pdm: 41.67 ± 4.972) and terminal branches (WT: 135.4 ± 11.13; WT + H1N1pdm: 90.03 ± 8.251; 5xFAD: 103.7 ± 8.238; 5xFAD + H1N1pdm: 44.8 ± 5.382), Sholl sum (WT: 2715 ± 245.9; WT + H1N1pdm: 1745 ± 140.1; 5xFAD: 2112 ± 160.4; 5xFAD + H1N1pdm: 1031 ± 81.37), and cellular surface area (WT: 143.5 ± 14.09 µm^2^; WT + H1N1pdm: 94.02 ± 7.519 µm^2^; 5xFAD: 111.9 ± 7.679 µm^2^; 5xFAD + H1N1pdm: 57 ± 3.899 µm^2^). These alterations were most pronounced in 5xFAD + H1N1pdm mice, which also exhibited a reduced longest shortest path (WT + H1N1pdm: 34.68 ± 2.431 µm; 5xFAD: 37.63 ± 2.229 µm; 5xFAD + H1N1pdm: 25.7 ± 1.353 µm) compared with non-infected 5xFAD mice. Together, these changes indicate a shift toward a less ramified and more compact microglial morphology, with the strongest morphological remodeling observed when H1N1pdm infection was combined with 5xFAD pathology.

## 3 Discussion

In the present study, we demonstrate that prior influenza A (H1N1)pdm infection modulates both cognitive and non-cognitive behavioral impairments in 5xFAD mice and is associated with pronounced synaptic and neuroinflammatory alterations. Importantly, our data indicate that viral infection acts as an independent pathological factor affecting brain function, while its interaction with established Alzheimer’s disease (AD) pathology is not strictly additive and may depend on the baseline state of neurodegeneration. Cognitive deficits are among the most robust and widely studied phenotypes in AD models, including 5xFAD mice. Consistent with the literature, we observed impairments in hippocampus-dependent spatial memory and associative learning. Previous studies indicate that 5xFAD mice typically develop spatial learning and memory deficits between 4 and 6 months of age, coinciding with amyloid deposition and neuroinflammation in hippocampal and cortical regions (Oakley et al., 2006; Kimura and Ohno, 2009; Jawhar et al., 2011; Webster et al., 2014; O'Leary et al., 2020). Our findings extend these observations by showing that prior viral infection alters behavioral outcomes, particularly affecting performance consistency and later phases of task execution, which may reflect impaired memory consolidation rather than acquisition alone. These findings are in line with previous reports demonstrating that peripheral infections can impair cognitive function and exacerbate neurodegeneration. Influenza A virus infection has been shown to induce long-lasting neuroinflammation, disrupt hippocampal synaptic plasticity, and impair spatial memory, even in the absence of direct viral invasion of the brain (Jurgens et al., 2012; Hosseini et al., 2018). Thus, the behavioral deficits observed in infected animals likely arise from inflammation-mediated disruption of hippocampal circuits rather than direct neurotropic effects of the virus.

In addition to cognitive impairments, our study highlights significant alterations in social and affective behaviors. We observed reduced social interaction and exploratory activity in 5xFAD mice, which were further modulated following viral infection. These findings are consistent with previous studies reporting social withdrawal and apathy-like behavior in 5xFAD mice, typically emerging around 6 months of age (Kosel et al., 2020; Jawhar et al., 2011; O'Leary et al., 2020). The additional impact of infection suggests that systemic inflammation may exacerbate dysfunction in limbic and prefrontal circuits involved in social behavior. We also observed alterations in exploratory behavior, including reduced rearing and changes in open field performance. However, interpretation of the OF results is complicated by the placement of both non-social and social stimuli in the center of the arena. Reduced investigation of these stimuli may therefore reflect anxiety-related avoidance of the central zone, reduced exploratory or social motivation, or a combination of these factors. For this reason, OF performance was not interpreted as an isolated measure of anxiety or social interest. Instead, these findings were considered together with complementary behavioral paradigms, particularly the three-chamber social test for social preference and the light–dark place preference test for anxiety-related behavior (Seibenhener and Wooten, 2015). Our results further demonstrate increased depressive-like behavior in 5xFAD + H1N1pdm mice, as evidenced by increased immobility in the tail suspension test (TST), particularly during the initial phase of the test. Notably, this effect was time-dependent and detected at the earliest measurement interval, while no differences were observed in total immobility. Furthermore, no significant changes were detected in the forced swim test (FST). This discrepancy may be attributed to methodological factors, particularly water temperature, which is known to critically influence behavioral and physiological responses in this paradigm. Previous studies have demonstrated that immobility in the FST is strongly dependent on water temperature and may reflect an adaptive thermoregulatory strategy rather than purely depressive-like behavior (Nadeau et al., 2022). Specifically, immobility is reduced or absent at lower temperatures, where animals display increased active escape-like behavior, likely to counteract heat loss (Nadeau et al., 2022). In line with this, variations in water temperature can significantly alter stress responsiveness, locomotor activity, and neurochemical signaling during the test (Linthorst et al., 2002). Under certain experimental conditions, lower or non-optimal temperatures may promote sustained escape-directed behavior instead of passive immobility, thereby masking depressive-like phenotypes. Thus, the absence of differences in the present study may reflect temperature-driven behavioral modulation rather than a true lack of affective alterations. However, this result is consistent with previous reports showing variability in depression-related phenotypes in 5xFAD mice (Kosel et al., 2020). Unexpectedly, 5xFAD + H1N1pdm mice displayed an anxiolytic-like phenotype in the light–dark place preference (LDPP) test. However, this was accompanied by reduced transitions between compartments, suggesting decreased exploratory activity or impaired behavioral flexibility rather than a true reduction in anxiety levels. Similar inconsistencies in anxiety-related behavior have been reported in 5xFAD mice across studies, highlighting the importance of cautious interpretation (Jawhar et al., 2011; O'Leary et al., 2020).

At the synaptic level, our electrophysiological data revealed a significant impairment of long-term potentiation (LTP) in 5xFAD mice compared to WT controls, consistent with previous studies demonstrating disrupted CA1 synaptic plasticity in this model (Kimura and Ohno, 2009; Rakovskaya et al., 2023). Following H1N1pdm infection, 5xFAD mice continued to exhibit reduced LTP relative to infected WT animals; however, no additional decrease in LTP magnitude was observed compared to non-infected 5xFAD mice. This indicates that viral infection does not further exacerbate already impaired hippocampal plasticity in this model. One possible explanation is that the hippocampus in 5xFAD mice is already in a state of chronic neuroinflammation, limiting the capacity for further synaptic deterioration in response to peripheral immune challenge (Rejc et al., 2022; Lin et al., 2023). This pattern may reflect a floor-like or occlusion effect, in which pre-existing synaptic pathology in 5xFAD mice reduces the dynamic range for detecting an additional infection-induced decline in synaptic plasticity. Several converging abnormalities in glutamatergic and Ca^2+^-dependent signaling may contribute to this apparent occlusion. Hippocampal LTP critically depends on tightly regulated postsynaptic Ca^2+^ signals initiated by glutamatergic receptors and coupled to downstream CaMKII-dependent plasticity mechanisms. Soluble Aβ oligomers have been shown to disrupt NMDA receptor-dependent signaling and promote dendritic spine loss (Shankar et al., 2007). In AD models, NMDA receptor-mediated Ca^2+^ influx can also trigger exaggerated ryanodine receptor-dependent Ca^2+^ release from the endoplasmic reticulum, thereby disturbing local dendritic and spine Ca^2+^ dynamics (Goussakov et al., 2010). In parallel, dysregulation of ER Ca^2+^ homeostasis and neuronal store-operated Ca^2+^ entry has been linked to reduced CaMKII activity, destabilization of mature mushroom spines, and impaired synaptic plasticity (Popugaeva et al., 2015; Zhang et al., 2015; Lacampagne et al., 2017). Restoration of STIM2-regulated Ca^2+^ entry has been shown to rescue both mushroom spine stability and hippocampal LTP deficits in AD models (Zhang et al., 2016). Together, these findings suggest that glutamatergic and Ca^2+^-dependent plasticity pathways may already be substantially compromised in 5xFAD mice, thereby limiting the measurable range for an additional H1N1pdm-induced reduction in LTP or dendritic spine parameters. Under these conditions, the additional inflammatory challenge may be expressed more prominently through enhanced glial reactivity and behavioral alterations. However, these signaling pathways were not directly assessed in the present study, and the proposed mechanism should therefore be considered a mechanistic interpretation requiring further experimental validation.

Importantly, the absence of LTP differences between WT and WT + H1N1pdm mice does not exclude functional effects of infection. Rather, the inflammatory response induced by the virus may not have reached the threshold required to produce persistent electrophysiological deficits at the time of analysis (Hosseini et al., 2018). Structural analysis of dendritic spines further supports these findings. 5xFAD mice exhibited a significant reduction in spine density and a shift toward immature spine types, characterized by decreased mushroom spines and increased thin and stubby spines. These changes are consistent with impaired synaptic stability and plasticity and are widely reported in AD models (Scheff et al., 2006; Shankar et al., 2007; Spires-Jones and Hyman, 2014). Consistent with this interpretation, individual spatial memory performance in the Morris water maze was positively associated with the proportion of mature mushroom spines, further supporting a relationship between dendritic spine maturation and hippocampus-dependent spatial memory. Notably, H1N1pdm infection alone induced similar alterations in WT mice, including reduced spine density and a shift toward immature spine morphology. However, consistent with LTP results, infection did not further exacerbate synaptic deficits in 5xFAD mice, supporting the idea that pre-existing synaptic pathology may reduce the dynamic range available for detecting an additional infection-induced synaptic decrement. Neuroinflammation likely represents a key mechanism underlying these effects. Both Western blot and immunohistochemical analyses revealed increased expression of GFAP and Iba1, indicating activation of astrocytes and microglia. In 5xFAD mice, glial activation was significantly elevated compared to WT controls, consistent with previous reports (Hong et al., 2016). Importantly, H1N1pdm infection further enhanced glial activation, particularly in 5xFAD mice, suggesting that systemic inflammation amplifies ongoing neuroinflammatory processes. Detailed morphological analysis further refined the characterization of glial reactivity. GFAP-positive astrocytes in 5xFAD mice exhibited an expanded cellular arbor and increased morphological complexity, including increased convex hull size, branching, Sholl complexity, and surface area. Interestingly, H1N1pdm infection did not produce a uniform additional increase in these astrocytic parameters, but selectively altered individual morphological features, indicating that astrocytic responses to the combined effects of AD pathology and systemic infection are not simply additive. In contrast, Iba1-positive microglia showed pronounced loss of ramification following H1N1pdm infection, characterized by fewer branch points and terminal branches, reduced Sholl complexity, shorter process extent, and reduced surface area, with the strongest remodeling observed in 5xFAD + H1N1pdm mice. These findings demonstrate that increased GFAP and Iba1 expression is accompanied by distinct structural remodeling of astrocytes and microglia and suggest that systemic infection differentially modifies glial morphology in the presence of established AD pathology. Importantly, GFAP and Iba1 expression together with morphological analysis characterize overall glial reactivity but do not distinguish molecularly defined astrocytic or microglial states. The absence of state- or subtype-specific molecular markers therefore remains a limitation of the present study. Activated microglia can contribute to synaptic loss through complement-mediated mechanisms, while reactive astrocytes can disrupt synaptic homeostasis (Hong et al., 2016; Woodburn et al., 2021). These processes provide a mechanistic link between infection-induced inflammation and the observed structural and functional synaptic deficits. In addition, H1N1pdm infection was associated with increased β-amyloid deposition in 5xFAD mice. Although the increase was modest, it suggests that systemic inflammation may influence amyloid dynamics, potentially through impaired clearance or altered processing pathways (Heneka et al., 2015; Cunningham, 2013). An important implication of our findings is the role of astrocytes as active regulators of synaptic function. Recent studies demonstrate that modulation of astrocytic activity can restore synaptic plasticity and improve cognitive performance in 5xFAD mice (Gerasimov et al., 2025; Lin et al., 2023; Gerasimov et al., 2023). This highlights astrocytes as a potential therapeutic target in conditions where neuroinflammation contributes to synaptic dysfunction.

Consistent with previous findings, our results demonstrate that 5xFAD mice exhibit profound behavioral, synaptic, and structural deficits associated with AD pathology. H1N1pdm infection of 5xFAD mice induces further neuroinflammatory and synaptic alterations and modulates behavioral outcomes; however, its impact on already impaired synaptic plasticity is limited, likely due to pre-existing synaptic impairment and the reduced dynamic range for detecting additional deterioration. These findings highlight the complex, non-linear interaction between neurodegeneration and systemic inflammation and emphasize the importance of considering disease stage when evaluating environmental risk factors in Alzheimer’s disease.

The present findings may have translational relevance for older patients with established Alzheimer’s disease who experience acute systemic viral infections. Clinical studies indicate that systemic infections and inflammatory episodes can accelerate cognitive decline in patients with AD, with deterioration persisting beyond the acute phase of infection (Holmes et al., 2009; Holmes et al., 2003). In patients with established AD, hospitalization and delirium are associated with increased risks of subsequent cognitive decline, institutionalization, and mortality (Fong et al., 2012), while broader clinical data indicate that dementia substantially increases vulnerability to adverse outcomes following infection (Naumova et al., 2009). More recent population-based data further show that individuals with AD experience a higher burden of infection-related hospitalization than matched individuals without AD, particularly for pneumonia and genitourinary infections (Järvinen et al., 2025). Importantly, influenza-specific preventive strategies may be clinically relevant in this vulnerable population: in a large nationwide cohort of older adults with dementia, influenza vaccination was associated with lower rates of hospitalization and mortality (Moses Appel et al., 2025). Our results suggest that infection-associated clinical deterioration may not reflect a uniform worsening of all AD-related pathological processes. Rather, systemic viral infection may preferentially amplify neuroinflammatory and behavioral alterations in the presence of already established synaptic pathology, consistent with reduced neural reserve in advanced neurodegeneration. From a clinical perspective, these findings support the importance of infection prevention, timely recognition and clinical management of systemic infections, and careful monitoring for post-infectious cognitive and behavioral decline in patients with AD. They also raise the possibility that therapeutic strategies aimed at limiting excessive neuroinflammatory responses or preserving synaptic resilience may be particularly relevant during or after systemic infection. However, these therapeutic implications remain hypothetical and require validation in prospective clinical and mechanistic studies.

## 4 Conclusion

In conclusion, this study demonstrates that prior influenza A (H1N1)pdm infection has long-lasting consequences for brain function and modifies the behavioral, synaptic, and neuroinflammatory phenotype of 5xFAD mice. The 5xFAD genotype was associated with pronounced impairments in hippocampus-dependent memory, social behavior, dendritic spine density and morphology, and CA1 synaptic plasticity. H1N1pdm infection induced additional behavioral alterations and enhanced glial activation, with the most pronounced neuroinflammatory response observed in infected 5xFAD mice. At the same time, infection did not further reduce LTP or spine density in 5xFAD animals, suggesting that established AD-like pathology may limit the magnitude of additional synaptic deterioration. These findings indicate that the interaction between systemic viral inflammation and neurodegeneration is not simply additive, but depends on the pre-existing pathological state and disease stage. Overall, our results support the view that peripheral viral infection can act as an important modifier of AD-related phenotypes and should be considered when evaluating environmental factors contributing to disease progression and clinical variability in older patients with cognitive impairment.

## 5 Methods

### 5.1 Virus

Mouse-adapted influenza strain A/California/7/09 (H1N1)pdm09 (H1N1pdm) was obtained from the Virus and Cell Culture Collection of the Smorodintsev Research Institute of Influenza in St. Petersburg, Russia. The strain was propagated in 11-day-old embryonated chicken eggs, purified using a sucrose gradient, and stored at −80 °C.

### 5.2 Animals

All animal procedures were conducted in compliance with the regulations for the ethical treatment of animals as outlined by the European Convention for the Protection of Vertebrate Animals Used for Experiments and Other Scientific Purposes (Strasbourg, 1986), and the ARRIVE guidelines. The study was approved by the Ethical Committee at the Smorodintsev Research Institute of Influenza, Russia (protocol № 62 dated 30.09.2022) and Bioethics Committee of the Peter the Great St. Petersburg Polytechnic University at St. Petersburg, Russia.

5xFAD (B6SJLF1/J background, Jackson Labs: strain #006554, Bar Harbor, ME, USA) and M-line (Thy1-GFP line M, Jackson Labs: stock #:007788) mice were used to create a transgenic 5xFAD-M line of mice. The breeding colony of 5xFAD and M-line mice was established and maintained in a vivarium with 4–5 mice per cage and a 12 h light/dark cycle in the animal facility. Transgenic mice from this line in the paper are called transgenic, and the WT mice from the same breed—M-line mice—are called wild-type mice (WT).

We confirmed 5xFAD or WT genotype using PCR method. For this purpose, DNA extraction from the tips of the tails was performed. We incubated the samples in alkaline buffer (25 mM NaOH, 250 µM EDTA, 75 µL) for 30 min at the temperature of 90 °C. Then, we added neutralizing buffer (40 мM Tris, pH = 4, 75 µL). Following primers were used for genotyping (Table 1). PCR was performed using 10 µL of 2xDreamTaq PCR mix; 3 µL of primer mix; 4,5 µL of DEPC-treated water; and 2,5 µL of a DNA sample. PCR condition contained initial denaturation (95 °C for 3 min), followed by 10 amplification cycles (94 °C for 15 s, 65 °C for 30 s, 68 °C for 45 s), and 28 amplification cycles (94 °C for 15 s, 65 °C for 30 s, 72 °C for 45 s). PCR products were visualized in 1% agarose gel by ethidium bromide (10 µg/mL). The presence of a single band indicates that the sample does not carry a transgenic mutation (WT), while two bands point out 5xFAD genotype.

**Table 1:** Primers for genotyping.

Mouse genotype	Primer	Sequence, 5’–3’
5xFAD	Forward	ACCCCCATGTCAGAGTTCCT
	Reverse	CGGGCCTCTTCGCTATTAC
M-line	Forward	ACAGACACACACCCAGGACA
	Reverse	CGGTGGTGCAGATGAACTT

### 5.3 Influenza infection

In the first stage, the minimum lethal dose of the virus (MLD_50_) was determined in 5xFAD and M-line mice. MLD_50_ represents the dose that causes the death of 50% of infected animals. The viral titers were calculated using the Reed-Muench method. The MLD_50_ value was found to be 10^–4.5^ TCID_50_/mL for both 5xFAD and M-line mice.

5xFAD and M-line mice were infected intranasally with H1N1pdm at the dose of 1 MLD_50_ per mouse. A volume of 30 µL of the viral suspension was administered, with the procedure performed under mild ether anesthesia. The mice were inhaled with 4–6% v/v diethyl ether. Control groups of mice (WT and 5xFAD) were treated with placebo (PBS); the administration was also performed under mild ether anesthesia. The mice were weighed daily, and their mortality and morbidity were monitored. After 2 weeks of infection, we formed following experimental groups: WT (*n* = 12), WT infected with H1N1pdm09 (*n* = 12), 5xFAD (*n* = 11), and 5xFAD infected with H1N1pdm09 (*n* = 8). We used these groups for further behavioral tests. All surviving animals included in these experimental groups underwent behavioral testing. After completion of the behavioral battery, animals were allocated to terminal experimental procedures depending on the type of downstream analysis. One subset was used for ex vivo hippocampal electrophysiological recordings and subsequent biochemical analysis, whereas a separate subset was perfusion-fixed and used for dendritic spine and immunohistochemical analyses. The numbers of biological samples, hippocampal sections, recordings, and dendritic segments used for each experimental procedure are specified in the corresponding Methods subsections and figure legends. A detailed scheme of the experiment is presented in Figure 1.

### 5.4 Open field (OF)

To test the level of anxiety in 5xFAD mice infected with the H1N1pdm09 virus, the three-stage OF test was carried out. The OF protocol was adapted from previously published work (Lopatina et al., 2014; Gerasimenko et al., 2020) and was used previously in our lab (Marinina et al., 2024a; Marinina et al., 2024b; Marinina et al., 2024c). The OF arena was made of an opaque, plexiglass chamber with a diameter of 62.4 cm subdivided into a center (with a diameter of 28.4 cm), intermediate and border (8.5 cm from chamber wall) zones, with aversive bright illumination (950 lx). During the first day of the OF test mice were positioned into the center of the arena and their behavior was recorded on video for 10 min. The following parameters were subsequently analyzed: time spent in the center, border, and the intermediate zones, time immobile, and frequency in the center zone. During the second day of the test a non-social novel object, consisting of a black metal mesh cylinder, was positioned in the middle of the OF arena. Mice were placed by the wall of the area at the beginning of the test and their behavior was recorded on video for 10 min. The following parameters were subsequently analyzed: time spent in the center, border, and the intermediate zones, total distance moved, exploration time, latency to novel object, and the number of transitions toward the novel object. During the third day of the OF test a social object, a foreign wild-type mouse of the same sex and age, was placed in the middle of the OF under the familiar cylinder. The test mouse was positioned by the wall of the OF arena at the beginning of the test and its behavior was recorded on video for 10 min. The following parameters were subsequently analyzed: time spent in the center, border, and the intermediate zones, total distance moved, time each mouse was exploring the cylinder with another mouse inside, latency to the object, and the number of visits toward the cylinder. The OF apparatus was cleaned between each trial with 70% ethanol. Each mouse underwent 1 trial. The results were analyzed via Any-maze software (Stoelting Europe, Ireland).

### 5.5 Three-chamber social test (3CST)

3CST was used to analyze social behavior of 5xFAD mice infected with the H1N1pdm09 virus. The 3CST protocol was modified from previously published work (Bohne et al., 2021) and was used previously in our lab (Marinina et al., 2024a; Marinina et al., 2024c). During this test, a 57 × 20 × 26 cm plexiglass chamber was divided into three equal sized chambers (19 × 20 × 26 cm) with equal openings (5 × 5 cm) to allow the open access to all three chambers. The arena was brightly illuminated with 950 lx. The first day of the test was used for habituation, experimental animals were positioned in the arena for 5 min. During the second day of the test, a non-social object, a black metal mesh cylinder (8 × 10 cm), was placed in the center of the left chamber (chamber “Cage”) and mice behavior was video recorded for 10 min. The following parameters were subsequently analyzed: time spent in the cage, start, and the empty zones, duration in the object zone (2 cm zone around the cylinder), and number of visits toward the cylinder. During the third day of the 3CST, a second black metal mesh cylinder containing a foreign mouse of the same sex and age was placed in the right chamber (chamber “Mouse”) and the test was repeated also for 10 min. The following parameters were subsequently analyzed: time spent in the cage, start, and the mouse zones, duration in the object zone (2 cm zone around the cylinder), number of visits toward the cylinder, duration in the social zone (2 cm zone around the cylinder with another mouse inside), and number of visits toward the cylinder with another mouse inside. We made sure that all the mice tested had total exploration time greater than 20 s and visited all three chambers during this test. The results were analyzed via Any-maze software (Stoelting Europe, Ireland).

### 5.6 Social interaction with resident intruder (SIWRI)

The SIWRI test was used to analyze social interaction of 5xFAD mice infected with the H1N1pdm09 virus. The SIWRI protocol was modified from previously published work (Wood and Morton, 2015; Bohne et al., 2021) and was used previously in our lab (Marinina et al., 2024a). Experimental animals were kept alone in a cage for 2 days before the test. During the experiment, each mouse was introduced to a foreign WT mouse of the same sex and age in a resident cage for 5 min. The experimental setup had a dim illumination (100 lx). The time duration and number of social events for fight (attack), social and genital sniffing were subsequently analyzed manually by an observer based on direct visual assessment according to predefined criteria. Social sniffing was determined as sniffing of the foreign mouse except its genitals. Genital sniffing was defined as sniffing of the intruder’s genitals. Environmental attention, environmental sniffing, resting, rearing, walking, following, mounting were also analyzed. Each mouse underwent 1 trial.

### 5.7 Forced swimming test (FST)

The FST was used to analyze mood decline in the 5xFAD mice infected with the H1N1pdm09 virus. During the FST each mouse was positioned in a glass beaker (15 cm height, 12 cm diameter) filled with water (21–23 °C) up to a height 12 cm. The beakers were placed in a chamber made in-house constructed of white laminated particle board for the top, base, sides, and dividers. The back panel was fabricated from white plexiglass. The test chamber (20 × 80 × 50 cm) was divided into four chambers (20 × 20 × 50 cm). The experiments were performed in a normal office lighting (400 lx) (Marinina et al., 2024c). After swimming for 6 min, the mice were transferred into a heated cage to recover and dry off. The amount of time that each mouse remained immobile was recorded via the Sony Cyber-shot DSC-W530 photo camera. In this test, the ‘immobile period’ was defined as the period when the animals stopped struggling for ≥ 2 s.

### 5.8 Tail suspension test (TST)

The TST was performed to study the depressive-lake state. The TST was carried out for a 6 min trial session. Mice were suspended 40 cm above the floor of a white chamber made in-house (20 × 80 × 50 cm). The experiments were performed in a normal office lighting (400 lx). The amount of time that each mouse remained immobile was recorded via the Sony Cyber-shot DSC-W530 photo camera. In this test, the ‘immobile period’ was defined as the period when the animals stopped struggling for ≥ 2 s.

### 5.9 Sucrose preference test (SPT)

The SPT was carried out to analyze the anhedonic-like behavior in 5xFAD mice infected with the H1N1pdm09 virus. Briefly, mice were given a choice between two bottles, one with 2% sucrose solution, and another one with water. Mice were habituated to the bottles for at least 2 days before the experiment. The trial was performed for 8 h. During the test no food was allowed to the mice under test. The position of the bottles was changed after 4 h to avoid the possibility of bottle location preference by experimental animals. The consumption of water and sucrose solution was analyzed by evaluation of liquid volume in the bottles prior to the test and in the end of the test. The preference for sucrose expressed as percentage was calculated as a ratio of the intake of the sucrose solution to the total fluid intake, multiplied by a hundred.

### 5.10 Light-dark place preference test (LDPP)

The LDPP test was carried out to further analyze the level of anxiety in 5xFAD mice infected with the H1N1pdm09 virus. The LDPP protocol was adapted from previously published work (Crawley and Goodwin, 1980) and was used previously in our lab (Marinina et al., 2024a). The test fixture consists of an open plexiglass box (20 × 26 × 28 cm) with aversive bright illumination (950 lx) with an opening into a smaller dark plexiglass box (9.5 × 15 × 9.5 cm) with no illumination. Experimental animals were placed in the light zone by the wall opposite to the dark zone entrance and their behavior was recorded for 5 min. The following parameters were subsequently analyzed: time spent in the light zone and number of zone transitions. The apparatus was cleaned between trials with 70% ethanol. Each mouse underwent 1 trial. The results were analyzed manually.

### 5.11 Morris water maze (MWM)

We conducted an evaluation of learning and memory impairments in mice infected with the H1N1pdm09 virus, using the MWM test. For this assessment, the mice were introduced to a circular pool (150 cm in diameter, 66 cm in height, with water maintained at 21–23 °C) surrounded by four visual cues placed at randomly assigned locations. The pool was filled with tap water made opaque by the addition of dry milk. A circular platform (10 cm in diameter, 27.5 cm in height) was submerged 1–1.5 cm below the water surface, serving as the escape platform. Over a period of 5 days, the mice underwent training to locate the hidden platform, with 4 trials conducted per day and a minimum inter-trial interval of 20 min. The starting position of each mouse was randomized across four cardinal directions, with the sequence remaining consistent for all mice throughout the training sessions. Each trial concluded either when the mouse successfully located the platform or after 90 s, at which point mice that failed to find the platform were gently guided to it. Mice were conditioned to remain on the platform for 15 s before being removed from the pool. Mice that did not locate the platform within the trial duration were placed on the platform for 15 s, held by the tail. On the fifth day, during the probe trial phase, the platform was removed, and mice were allowed to swim freely for 90 s. The performance of each mouse during the trials was meticulously recorded using ANY-maze automated behavioral video tracking software, version: 7.49 (Stoelting Europe, Dublin, Ireland).

### 5.12 Fear conditioning (FC)

Contextual and cued fear memory was assessed via the FC test. To eliminate potential freezing behavior due to the novelty of the environment, the mice were acclimated to the chamber prior to conditioning. On day 1 (habituation), mice were individually introduced into the habituation room and placed in the training chamber for 5 min without any auditory or tactile stimuli. The next day the training phase was carried out. Mice were positioned in a training chamber consisting of transparent plexiglass walls (20 × 26 × 28 cm) with a stainless-steel grid floor (0.2 cm diameter, spaced 0.5 cm apart) and were allowed to explore freely the cage for 2 min. The arena had a dim illumination around 100 lx. Subsequently, a loud tone (conditioned stimulus, CS, 55 dB white noise) was presented for 20 s, followed by a mild foot shock (1.5 s, 0.5 mA, unconditioned stimulus, US) with two subsequent repeats to force the learning. The percentage of freezing during the training was assessed. The next day the context testing was carried out. The mice were positioned into the same cage and their contextual memory was assessed for 3 min. At least an hour later that day mice were positioned into a new cage with different conditions such as white acrylic sides and floor, different lightning (950 lx) and odor to analyze their cued fear memory. The mice were allowed to explore the new cage for 3 min. Then the loud tone was presented for 3 min and the cued fear memory was assessed. Mice were video tracked via the Logitech C922 Pro Stream Webcam positioned above the arena and the Logitech WebCam C200 positioned to the side of the arena. The apparatus was cleaned between trials with 70% ethanol. Freezing time was calculated using ANY-Maze tracking software version 7.49 (Stoelting Europe, Dublin, Ireland). The context and cued long-term memory were analyzed on the tenth day.

### 5.13 Long-term potentiation recording

For the long-term potentiation experiments, C57/BL/6 (WT) and 5xFAD control mice, and mice that had recovered from H1N1pdm infection were used. At 8 months of age, ex vivo hippocampal slices were prepared from the mice, and field excitatory postsynaptic potentials (fEPSPs) were recorded to assess the level of long-term potentiation. For this purpose, mice were anesthetized with isoflurane (1.5-2%) and perfused transcardially with ice-cold modified holding artificial cerebrospinal fluid (holding ACSF) containing (in mM) 12.5 D-glucose, 5 HEPES, 124 NaCl, 2.5 KCl, 3 Na-pyruvate, 1.25 NaH2PO4·2H2O, 24 NaHCO3, 0.5 CaCl2·2H2O, and 10 MgSO4·7H2O (pH 7.3–7.4). Brains were rapidly removed into ice-cold modified holding ACSF. The 400 μm thick slices were cut on a vibratome (Leica Microsystems, Wetzlar, Germany) and immediately transferred to an incubation chamber (BIOSAN WB-4MS, Riga, Latvia). The slices were incubated for recovery at 32 °C for 30 min, after which they were transferred to incubation chamber containing recording ACSF with (in mM) 12.5 D-glucose, 5 HEPES, 124 NaCl, 2.5 KCl, 1.25 NaH2PO4·2H2O, 24 NaHCO3, 2 CaCl2·2H2O, and 0.8 MgSO4·7H2O (pH 7.3–7.4) for 60 min at room temperature. During the slice preparation and recording, artificial cerebrospinal fluid was continuously mixed with the gas, including 95% O2 and 5% CO2. After incubation at room temperature, the hippocampal slice was transferred to a recording chamber with a continuous supply of recording ACSF via peristaltic pumps LONGER BQ50-1J-A (Longer Precision Pump Co., Ltd., Baoding City, China) and left for 20 min to reach a steady state before fEPSP recording began.

The stimulating bipolar electrode was prepared by double-twisting two platinum-iridium wires with a diameter of 50 µm (A-M Systems Inc., Carlsborg, WA, USA). The recording electrode consists of a silver wire (A-M Systems Inc., Carlsborg, WA, USA) that was chlorinated in a 1 M KCl solution, then the electrode was inserted into a borosilicate pipette (Sutter Instrument, Novato, USA) fabricated using a Micropipette Puller P-1000 (Sutter Instrument, Novato, USA). The stimulation site was selected in the region located at the border between the CA1 and CA2 areas of the hippocampus, with the recording site determined by the appearance of an fEPSP response in the CA1 area. Using a Sutter Instrument MPC-200 micromanipulator (Sutter Instrument, Novato, USA), the stimulating and recording electrodes were inserted into the slice to a depth of 80–100 µm. The fEPSPs were evoked by a Model 2100 isolated pulse stimulator (A-M Systems Inc., Carlsborg, WA, USA), with the stimulation current (starting at 10 μA in 5–10 μA increments) adjusted until a population spike appeared. The evoked fEPSPs were amplified with a MultiClamp 700B (Molecular Devices, San Jose, CA, USA) and digitized with a Digidata 1440A (Molecular Devices, San Jose, CA, USA) at a 20 kHz sampling rate. Baseline responses were stabilized (fEPSP slope deviation of less than 10%) and then recorded for 20 min at 0.05 Hz of stimulation. Long-term potentiation was then induced by high-frequency stimulation (HFS), which consisted of two bursts with an interval of 20 s; each burst had a duration of 1 s and a stimulation frequency of 100 Hz. After HFS, the stimulus was repeatedly delivered once every 20 s for 60 min to observe any changes in the long-term potentiation (LTP) magnitude. The LTP magnitude was defined as the ratio between the average fEPSP slope after HFS during the last 10 min and the average slope of the baseline fEPSPs. Traces were obtained and analyzed using the pClamp 10.7 software program (Molecular Devices, San Jose, CA, USA).

### 5.14 Evaluation of density and morphology of dendritic spines

The preparation of fixed slices of the hippocampus brain region was performed after transcardial perfusion with 1.5% PFA (pH = 7.4) solution in PBS. Mice were anesthetized via urethane (200 μL of a 250 mg/mL solution) diluted in a 0.9% NaCl solution. Hippocampal sections (50 μm thick) were obtained using a vibrotome (5100mz Campden Instruments; Leicestershire, England) and incubated in 0.5% PFA at 4 °C. For microscopy slices were glued with Aqua Poly/Mount (Polysciences, #18606, Warrington, PA, USA) on microscope slides (Heinz Herenz, # 1042050, Hamburg, Germany) and coverslips (Menzel Glaser, #1, Braunschweig, Germany). Images of secondary dendrites of hippocampal neurons were made by a ThorLabs confocal microscope with the following parameters: Olympus UPlanSApo × 100 lens (UPlanSApo, 100×/1.40 Oil, OLYMPUS, Tokyo, Japan), resolution 2048 × 2048, slice thickness 0.2 µm/pixel. After that, secondary dendritic spines density and morphology were analyzed at Neuronstudio (Rodriguez et al., 2008). For analysis, a segment of the dendrite was selected, within which the total number of spines and the number of spines by type were quantified. The obtained values for each dendritic segment were normalized to its length, which was subsequently standardized by recalculating all data to a 10 μm dendritic length to enable comparison between groups. Dendritic spine density was defined as the number of spines per unit length of the analyzed dendrite. Dendritic spine analysis included 20, 12, 20, and 8 hippocampal sections from the WT, WT + H1N1pdm, 5xFAD, and 5xFAD + H1N1pdm groups, respectively. A total of 100, 60, 100, and 40 dendritic segments, respectively, were analyzed.

### 5.15 Western blot

The hippocampus was extracted from both hemispheres. Hippocampal lysates were separated using 5% polyacrylamide stacking and 4% resolving gels under denaturing conditions, then transferred to a polyvinylidene fluoride (PVDF) membrane (#IPVH00010, Merck Millipore) in transfer buffer (25 mM Tris, 192 mM glycine, 20% methanol (#67-56-1, Merck Millipore), 0.01% SDS) at 25 V for 30 min (BioRad). The membrane was blocked for 1 h in 5% BSA/TBST (tris-buffered saline with 0.1% Tween 20: 50 mM Tris, 150 mM NaCl, 0.1% Tween 20, pH = 7.6). After blocking, the membrane was incubated overnight (16 h) with primary antibodies to Iba-1 (1:1000, Cell Signaling Technolody – 17198S), to GFAP (1:10000, BioLegend – 840001) and to Actin (1:2000, Millipore - MAB1501). The membrane was then washed and incubated with secondary antibodies conjugated with horseradish peroxidase anti-rabbit antibody (1:2000 dilution, DAKO - P0448), or anti-mouse antibody (1:2000 dilution, DAKO - P0447. Next, the membrane was washed several times and incubated in a solution that induces a chemiluminescence reaction. The fluorescent signal was detected using X-ray film (CP-BU NEW, #EWPJH, AGFA) according to the manufacturer's recommendations. Western blot images were analyzed using ImageJ software. The expression level of Actin was used as a control for protein loading in the sample. All data were normalized within each staining to the control group's mean inoculated with PBS. Western blot analysis was performed using hippocampal samples from four mice per experimental group.

### 5.16 Immunohistochemistry

Brain sections from 9-month-old mice of four experimental groups (WT, WT + H1N1pdm, 5xFAD, 5xFAD + H1N1pdm) were placed in a 24-well tablet in 0.5% PFA overnight. The next day, immunohistochemical staining was performed. First, the sections were washed twice for 10 min in PBS, then incubated in 20% HCl solution to reduce autofluorescence and improve antibody binding, followed by washing in 0.1% Tween-20. The plate was kept on a shaker throughout all procedures. Next, the sections were incubated in a blocking solution containing 5% bovine serum albumin (BSA) and 0.1% Triton X-100 in PBS for 1 h at room temperature (RT). After that, the sections were incubated overnight at 4 °C with primary antibodies: mouse monoclonal anti-Aβ40 (1:1000, Servicebio - SB-GB121197), rabbit polyclonal anti-Iba1 (1:500, Cell Signaling Technolody – 17198S), and rabbit polyclonal anti-GFAP (1:1000, BioLegend – 840001), diluted in a solution containing 5% BSA and 0.125% Triton X-100 in PBS. The next day, the sections were washed three times in PBS for 10 min and then incubated at RT for 1.5 h with secondary antibodies Alexa Fluor 594 Goat anti-Rabbit IgG (H + L) (1:1000, Invitrogen - R37117) and Alexa Fluor 594 Goat anti-Mouse IgG (H + L) (1:1000, Invitrogen - R37121), diluted in a solution containing 5% BSA and 0.125% Triton X-100 in PBS. After washing three times in PBS, the stained sections were mounted under coverslips using Aqua Poly/Mount mounting medium. Immunohistochemical analysis included 10, 8, 10, and 6 hippocampal sections from the WT, WT + H1N1pdm, 5xFAD, and 5xFAD + H1N1pdm groups, respectively. These sections were obtained from 5, 4, 5, and 3 mice, respectively.

### 5.17 Imaging and quantification of cell density

Brain slices were visualized using a confocal microscope (ThorLabs, USA) with a × 4 objective lens. Image parameters included a resolution of 2,048 × 2,048 pixels, 1,156 μm/pixel, and a step size along the *z*-axis of 5 μm. Imaging of fixed samples was performed in the red fluorescence channel (excitation wavelength 594 nm). Images were analyzed using the ImageJ software, and areas of positive cells were identified using a bandpass filter (with uniform settings for all images), automatic binarization (with a universal threshold for all images), and the “Analyze Particles” function. The number of cells and the area occupied by them, normalized per unit area, were considered in the calculations. All data were normalized within each staining to the control.

For detailed glial morphology analysis, high-magnification images of individual GFAP-positive astrocytes and Iba1-positive microglia were acquired using a × 100 objective lens. Image parameters included a resolution of 2,048 × 2,048 pixels, 0.046 μm/pixel, and a step size along the z-axis of 0.3 μm. Individual cells were reconstructed and analyzed using Fiji software, and morphological analysis was identified using a bandpass filter (with uniform settings for all images), automatic binarization (with a universal threshold for all images), and the “Neuroanatomy” plugin. For GFAP-positive astrocytes, branch length, convex hull size, number of branch points, Sholl sum, and surface area were quantified. For Iba1-positive microglia, longest shortest path length, number of branch points, number of terminal branches, Sholl sum, and surface area were quantified.

### 5.18 Statistical analysis

Statistical analyses were conducted using GraphPad Prism 9 (GraphPad Software, Version 9.0). Data distribution was assessed using the Shapiro–Wilk test. For normally distributed data, homogeneity of variances was assessed using Bartlett’s test, where applicable. Comparisons between two groups were performed using Student’s *t*-test. For comparisons involving more than two groups with a single experimental factor, one-way ANOVA followed by Tukey’s post-hoc test was used. For datasets involving the four experimental groups (WT, WT + H1N1pdm, 5xFAD, and 5xFAD + H1N1pdm), two-way ANOVA was performed with genotype (WT vs. 5xFAD) and infection status (control vs. H1N1pdm) as independent factors. The main effects of genotype and infection, as well as the genotype × infection interaction, were evaluated. When a significant interaction effect was detected, post-hoc multiple comparisons were performed using Tukey’s test. For data that did not follow a normal distribution, comparisons between two groups were performed using the Mann–Whitney U test, whereas comparisons among more than two groups were performed using the Kruskal–Wallis test followed by Dunn’s multiple-comparisons test. Associations between behavioral measures and subsequent morphological, electrophysiological, or biochemical parameters obtained from the same individual animals were assessed using Spearman’s rank correlation analysis. Correlation coefficients are reported as Spearman’s *r*_s_ with two-tailed *p* values. Only animals for which both variables of a given correlation were available were included in the corresponding analysis. Sample sizes are reported for each experimental endpoint in the corresponding Methods subsection or figure legend. No formal a priori power calculation was performed. Sample sizes were based on previous studies using comparable behavioral, electrophysiological, morphological, and biochemical approaches in 5xFAD mice, together with tissue availability following behavioral testing and animal-welfare considerations in accordance with the reduction principle. The exact statistical test used for each analysis is specified in the corresponding figure legend. Survival curves were analyzed using the Mantel–Cox test. p-values for statistical significance are provided in the figure legends.

## Data Availability

The original contributions presented in the study are included in the article/Supplementary material, further inquiries can be directed to the corresponding author.
