# Supplementary material for: Influenza A (H1N1)pdm infection exacerbates behavioral, cognitive, and synaptic impairments in 5xFAD mice
Source: Front Behav Neurosci. 2026 Oct 8;20:1955816. doi: 10.3389/fnbeh.2026.1955816 (PMC13687074; doi:10.3389/fnbeh.2026.1955816)
Supplement: Supplementary file 1 [file Data_Sheet_1.PDF]

Supplementary materials

A

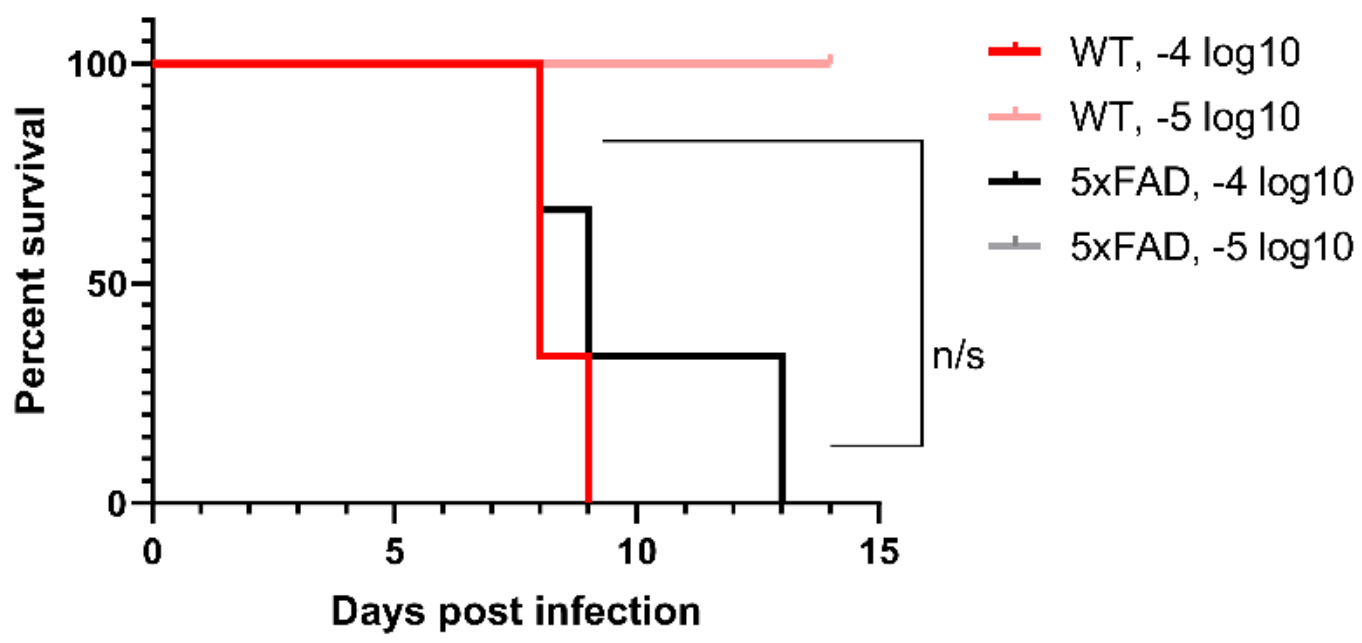

B

Minimum lethal dose of the virus (MLD_{50}) for 5xFAD and WT mice

Line	5x FAD	WT
$log_{10}(TCID_{50})$/mL	4.5	4.5

Figure S1. (A) Female 5xFAD and WT mice were infected with H1N1pdm09 in tenfold viral dilutions. Data are presented as Kaplan–Meier survival curves. Diluted in 105 times (-5 log10) virus did not cause lethal cases in both lines. In tenfold smaller dilution (-4 log10) of H1N1pdm09, all the mice succumbed to the infection. We did not notice significant difference in survival curves between 5xFAD and WT in any viral dilution, statistical analysis was performed using the Mantel-Cox test; n/s – no statistical difference. (B) We determined minimum lethal dose of the virus (MLD50) that causes the death of 50% of infected animals. Using the Reed-Muench method, viral titers were calculated. According to our results, the dose was the same for both mice lines and was 4.5 log10(TCID50)/mL. Therefore, dilution of the initial viral stock in 104.5 times (approximately 31623 times) causes lethal outcomes in a half of 5xFAD or WT mice.

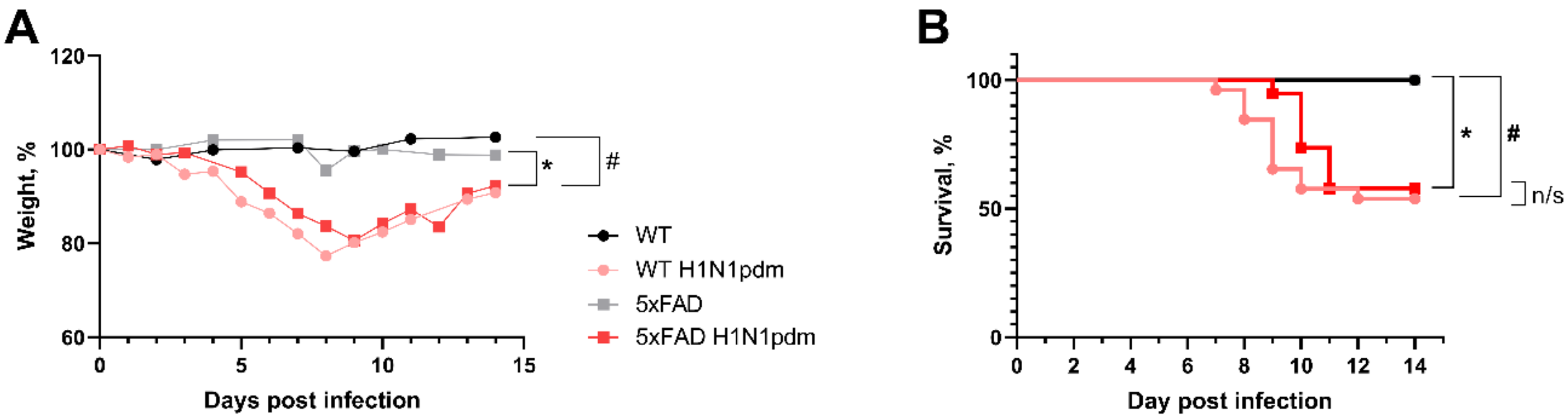

Figure S2. H1N1pdm09 caused significant morbidity and mortality in both 5xFAD and WT mice. (A) Dynamics of body weight changes in control or infected with H1N1pdm09 mice. Influenza infected WT (light red, circles, N = 23) and 5xFAD mice (red, squares, N = 13) were characterized by reduced body weight in comparison with mock infected WT (black, squares, N = 12) and 5xFAD (black, circles, N = 11) mice, respectively. Data are presented as mean ± SEM. *$p < 0.05$ in relation to mock infected 5xFAD; #$p < 0.05$ in relation to mock infected WT, statistical analysis was performed using repeated measures one-way ANOVA with Tukey's multiple comparisons test. (B) Survival curves of infected 5xFAD and WT mice compared with mock infected controls. Infection with H1N1pdm09 resulted in 5xFAD or WT in about half of cases. Influenza infected 5xFAD and WT mice did not differ in lethality. Data are presented as Kaplan–Meier survival curves. *$p < 0.05$ in relation to mock infected 5xFAD; #$p < 0.05$ in relation to mock infected WT; n/s – no statistical difference. Statistical analysis was performed using the Mantel-Cox test, with Bonferroni correction for multiple comparation.
